# Is Vitamin D Deficiency the Cause or the Effect of Systemic Lupus Erythematosus: Evidence from Bidirectional Mendelian Randomization Analysis

**DOI:** 10.1155/2022/8689777

**Published:** 2022-09-21

**Authors:** Yang Ding, Shengyi Yang, Shasha Fan, Yi Tang, Yan Teng, Xiaohua Tao, Wei Lu

**Affiliations:** ^1^Health Management Center, Department of Dermatology, Zhejiang Provincial People's Hospital, Affiliated People's Hospital, Hangzhou Medical College, No. 158, Shangtang Road, 310014 Hangzhou, China; ^2^Key Laboratory of Environmental Medicine Engineering, Ministry of Education, Department of Epidemiology & Biostatistics, School of Public Health, Southeast University, Nanjing, China

## Abstract

**Background:**

Vitamin D deficiency is common in patients with systemic lupus erythematosus (SLE). Observational studies have reported that it is associated with SLE. In this bidirectional Mendelian randomization (MR) study, we explored the genetic association between serum vitamin D (VD) levels and SLE using two models.

**Methods:**

Genetic variants associated with vitamin D (*n* = 304,181), 25-hydroxyvitamin D levels (*n* = 401,460), and SLE (*n* = 213,683) at genome-wide significance (*P* < 5∗10^−8^) derived from large-scale publicly available GWAS data were used as instrumental variables. Bidirectional two-sample MR analyses were performed using the inverse variance weighted method (IVW, random, or fixed effect model). Sensitivity analyses including maximum likelihood, MR-Egger method, penalized weighted median method, MR-PRESSO, MR-RAPS, and MR-radial method were conducted.

**Results:**

The findings showed that genetically predicted SLE using the IVW method had a negative effect on the vitamin D and 25-hydroxyvitamin D levels in the two models. The results of sensitivity analyses of different analytical approaches were consistent.

**Conclusions:**

These findings indicated that genetically determined SLE had a negative effect on the vitamin D and 25-hydroxyvitamin D levels. Future studies, including random controlled clinical trials, should evaluate the association and mechanisms between serum VD levels and SLE.

## 1. Background

Systemic lupus erythematosus (SLE) is a kind of disease characterized by aberrant activity of the immune system leading to chronic damage in relative organs [1]. Relevant statistical results show that the incidence of SLE is higher in women of childbearing age, and the female to male incidence ratio is approximately 13 : 1 [2]. SLE is a major health burden globally; therefore, early prognosis and effective preventive approaches are urgently needed.

Vitamin D is a vital steroid hormone and antifibrotic effects [3]. Vitamin D is converted to 25-hydroxyvitamin D which is used as an indicator. Vitamin D has been reported to be associated with SLE in observational studies. This implies that vitamin D deficiency may be a risk factor for SLE. Previous systematic reviews and meta-analysis indicated that levels of serum VD [4] and 25-hydroxyvitamin D levels [5] (the index for measuring vitamin D level) are obviously low in SLE patients. However, findings from observational studies are not sufficient to make clear this kind of relationships, which are explored through reverse causation and confounding effect [6]. A MR analysis showed that vitamin D deficiency has no relation with SLE [7]. Therefore, whether vitamin D deficiency is the cause or the effect of SLE has not been fully elucidated.

MR analysis using genetic variants as instrumental variables (IVs) to identify the causation between risk factors and diseases [8, 9]. In this paper, a bidirectional MR study was performed to explore the genetic association of serum VD levels and SLE using large-scale publicly available GWAS data.

## 2. Materials and Methods

### 2.1. Data Retrieval for MR Analyses

**Figure 1:**
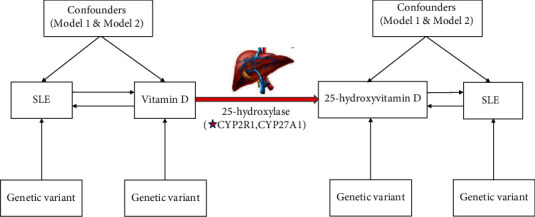
Overview of the experimental design of the present study. Model 1: SNPs associated with any potential confounders were not extracted. Model 2: SNPs associated with any potential confounders were extracted. SLE: systemic lupus erythematosus.

Two models were utilized to perform bidirectional MR analyses to explore the potential causal associations of vitamin D and 25-hydroxyvitamin D with SLE using summary statistics data from four different genome-wide association studies (GWAS) (Figure 1). Model 2 was established by extracting SNPs on the outcome, whereas in model 1, SNPs were kept. Summary statistical data were retrieved from meta-analysis GWAS of 35 biomarkers deposited in the UK Biobank (UKB) [10]. The UK Biobank was a cohort study involving more than 500,000 males and females (40–69 years of old at baseline) enrolled between 2006 and 2010 [11]. Publicly available summary-level data for 25-hydroxyvitamin D were obtained from GWAS. The participants in this cohort presented with SNPs with minor allele frequency (MAF) > 0.1% and were adjusted for age, sex, and VD [12]. SLE data were obtained from a previous meta-analysis of GWASs with 10,000 subjects, when SLE was used as exposure factor, covering approximately 644,000 markers [13]. SLE data were obtained from GWAS involving 213,683 European-ancestry subjects deposited in FinnGen biobank, when SLE was used as an outcome. The details on the data used in the current study are presented in Table 1.

### 2.2. Selection of Genetic Instrumental Variables

Genetic variants used in the MR analyses were of genome-wide significance (*P* < 5 × 10 − 8) and were distributed independently by pruning SNPs with an *r*^2^ < 0.001 threshold [14]. SNPs associated with body mass index, C-reactive protein, and leukocyte count were identified as pleiotropic IVs and were extracted from the GWAS Catalog and PhenoScanner, to eliminate potential pleiotropic effects [15]. Subsequently, exposure-related SNPs were obtained from the outcome datasets. In order to correct the direction of alleles, SNP coordination was carried out after appropriate simplification in the research process. In total, 53 SNPs (model 1) and 46 SNPs (model 2) were selected as instrument on SLE. In addition, 160 SNPs (model 1) and 147 SNPs (model 2) were selected for 25-hydroxyvitamin D on SLE, as well as 8 SNPs (model 1) and 7 SNPs (model 2) for SLE on vitamin D. Moreover, 38 SNPs (model 1) and 33 SNPs (model 2) were selected for SLE on 25-hydroxyvitamin D levels. The F statistics for each instrument-exposure effect were above 10, indicating a low risk. The formula of F statistic was as follows: *F* = [(*N* − *K* − 1)/*K*]∗[*R*^2^/(1 − *R*^2^)], where *R*^2^ indicates the rate of variance according to: *R*^2^ = 2∗(1 − minimum allele frequency (MAF))∗MAF∗(Beta/stand error)^2^. Details on selection of variables are presented in Table 1 and supplementary material 1-8.

### 2.3. Mendelian Randomization Estimates

A bidirectional two-sample MR was conducted to explore the casual effect of VD levels and SLE. The IVW method was used to pool Wald ratio estimates of SNPs in the primary analysis [16]. In this regard, the IWW method is widely used in the statistical analysis process. The application of IVW presupposes that all SNPs are valid IVs; thus, this method can help achieve accurate estimation results. If there is no evidence of directed pleiotropy in the selected IVS, it can be considered that the results obtained by this method are very reliable and meet the relevant analysis requirements [17].

Further, complementary analyses were conducted using MR-egger method [18], weighed median method [19], maximum likelihood method [16], and penalized weighted median method. The MR-Egger method requires regression analysis during processing [18]. Weighted median estimator can make out the relative causal effect, if more than half of the SNPs are valid IVs [19]. MR-PRESSO analysis was conducted to verify the results obtained by IVW. MR-PRESSO detects the effects of outliers and horizontal pleiotropy [20]. IVW radial variant models were established and used to regress the product of the Wald ratio estimate. In addition, this method can also be used to evaluate the weighted square root of genetic variation, which can obtain better IVW results, and also provide support for subsequent visual processing, so it is widely used in this field [21].

### 2.4. Heterogeneity and Pleiotropy Analysis

Cochran's *Q* method can be used to analysis the heterogeneity. The final MR results were evaluated using a multiplicative random-effects model of IVW with a *P* value of Cochran's *Q* test 0.05 [22]. Further analysis was conducted to control or correct directional pleiotropy. The MR-Egger intercept was performed to test for bias attributed to directional pleiotropy. The results of statistical analysis showed that the average influence of the tested gene variant was nonzero, and the influence of the average pleiotropy corresponded to the intercept [18]. In the process of simulation analysis, mr-raps was used to simulate the distribution of pleiotropic effects of genetic variation, and the results were compared to provide support for subsequent analysis [23]. In addition, MR-PRESSO method was used to conduct a global test of heterogeneity and for identification of horizontal pleiotropy. Further, leave-one-out sensitivity analysis was performed to assess if the causal association was driven by a single SNP.

Analyses were conducted by R software using “Two-Sample-MR,” “MR-PRESSO,” “Radial MR,” and “MR-RAPS” packages. All statistical tests were two-sided, and *P* value < 0.05 was defined as statistical significance.

## 3. Results

### 3.1. Causal Role of SLE on Serum Vitamin D and 25-Hydroxyvitamin D Levels

**Figure 2:**
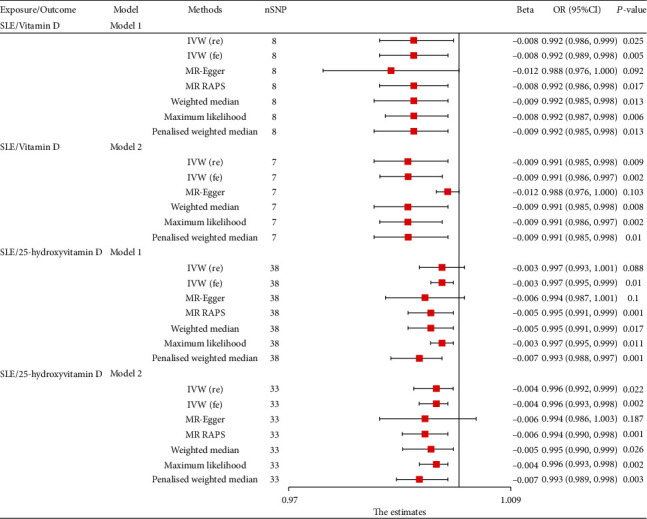
MR results for causal role of systemic lupus erythematosus on serum vitamin D and 25-hydroxyvitamin D levels. MR-RAPS method was not applicable for causal effect from SLE on vitamin D with model 2 for limited SNPs. SLE: systemic lupus erythematosus; SNP: single-nucleotide polymorphisms; IVW: inverse variance weighted; MR-RAPS: MR-robust adjusted profile score.

Using the Mendelian randomization with fixed-effect IVW method, we found that genetically predicted SLE was significantly associated with decreased levels of vitamin D in model 1 (*P* = 0.005) and model 2 (*P* = 0.002, Figure 2). The causal estimates were comparable among the implemented MR methods except the MR-Egger method (Figure 2). Some outliers were identified using radial plots (Supplementary Figure 2-3) but no outliers were identified through MR-PRESSO method (Table 2). The raw estimates obtained by MRPRESSO indicated that genetically predicted SLE has strong relationship with decreased levels of vitamin D (model 1: *P* = 0.047; model 2: *P* = 0.041). MR-RAPS was performed to explore whether vitamin D affected SLE through several weak instruments. MR-RAPS results showed that vitamin D levels had a causal effect on SLE in model 1 (*P* = 0.017, Figure 2). Diagnostic plots generated by MR-RAPS are presented in Supplementary Figure 3-4. Results of the MR-Egger analysis showed no indication of directional pleiotropy (*P* = 0.396). Moreover, there was no obvious heterogeneity among SNPs (*P* = 0.148 and 0.223, respectively, Supplementary Table 9). No individual SNP contributed significantly to the relation between SLE and level of vitamin D.

**Figure 3:**
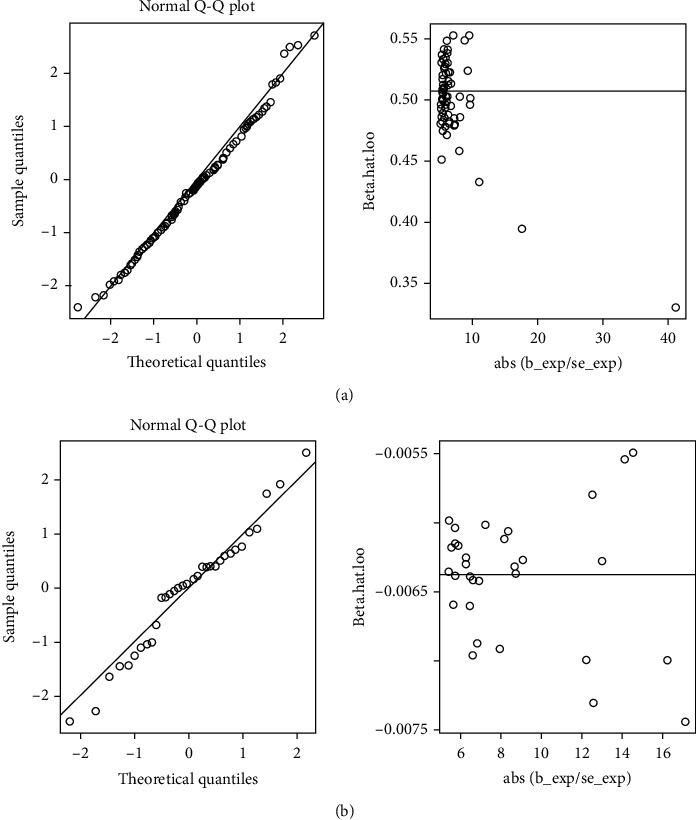
Diagnostic plots generated by MR-RAPS. (a) Causal role of SLE on 25-hydroxyvitamin D levels (model 1). (b) Causal role of SLE on 25-hydroxyvitamin D levels (model 2). SLE: systemic lupus erythematosus.

Genetically predicted SLE was associated with 25-hydroxyvitamin D as shown by the random-effect IVW analysis after removing confounder-related SNPs (model 2: OR: 0.996, 95% CI: 0.992–0.999, *P* = 0.022, Figure 2). The causal estimates were consistent for all implemented MR methods except MR-Egger method (Figure 2). Outliers were identified in model 1 and model 2 using radial plots (Supplementary Figure 5-6) and MR-PRESSO method (Table 2). Outlier corrected estimates obtained by MRPRESSO were consistent with the results from IVW method. Potential heterogeneities were observed, but the results did not show directional pleiotropies (Supplementary Table 9). Diagnostic plots generated by MR-RAPS are presented in Figures 3(a) and 3(b).

### 3.2. Causal Role of Serum Vitamin D and 25-Hydroxyvitamin D Levels on SLE

**Figure 4:**
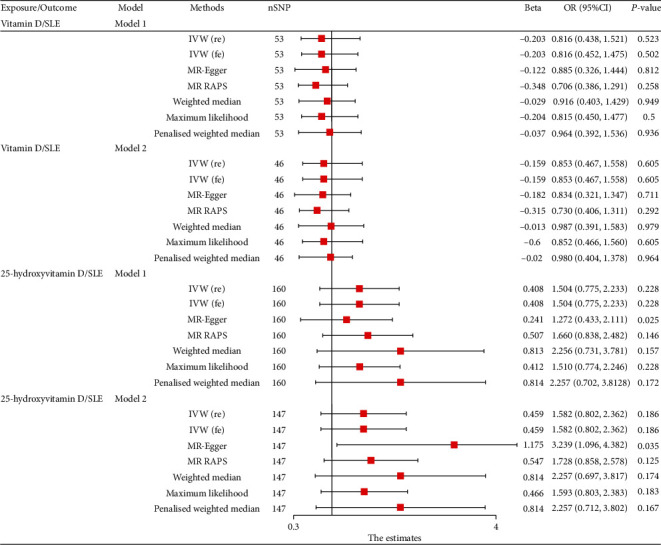
MR results for causal role of serum vitamin D and 25-hydroxyvitamin D levels on SLE. MR-RAPS method was not applicable for causal effect of SLE on vitamin D with model 2 for limited SNPs. SLE: systemic lupus erythematosus; SNP: single-nucleotide polymorphisms; IVW: inverse variance weighted; MR-RAPS: MR-robust adjusted profile score.

Results from fixed-effect IVW analysis indicated that low vitamin D levels were not associated with a high risk of SLE in model 1 (*P* = 0.502) and model 2 (*P* = 0.605, Figure 4). The causal estimates were consistent for all implemented MR methods (Figure 4). MR-PRESSO was conducted to evaluate the raw estimates, and the results showed similar results that VD levels had no causal effect on the risk of SLE in model 1 (*P* = 0.299) and model 2 (*P* = 0.337). The findings indicated no potential heterogeneity and then bring in relevant data to statistically analyze the directional pleiotropy of the results (Table 9). Radial plots obtained through MR-radial method and diagnostic plots obtained by MR-RAPS are presented in Supplementary Figure 7-10. Leave-one-out result showed that there is no single SNP associated with the correlation between the two.

The findings revealed no causal effect of serum 25-hydroxyvitamin D levels on SLE (Figure 4). Notably, the causal estimates were consistent in all implemented MR methods except MR-egger method (Figure 4). The results showed no potential heterogeneities among the results. Radial plots obtained by MR-Radial and diagnostic plots generated by MR-RAPS are presented in Supplementary Figure 11-14.

## 4. Discussion

The current study presents the first bidirectional MR to research the genetic association of serum VD levels and SLE using two models. We found that genetically determined SLE was causally associated with low vitamin D levels.

Vitamin D affects physiological systems in addition to its function in bone homeostasis [24]. Studies have reported that vitamin D signaling can significantly affect biological processes related to immune response, and the corresponding mechanism is very complex [25]. For instance, the active form of vitamin D and 1,25-dihydroxyvitamin D has potent immunomodulatory effects. SLE is a multisystem autoimmune disease. A potential association between VD and SLE has been demonstrated previously. However, the type of correlation between them has not been fully elucidated [26, 27, 28]. Furthermore, majority of evidence supporting the current understanding on this association is from observational studies, and not study has explored the reverse causality. A previous meta-analysis demonstrated that SLE patients had significantly low serum levels of vitamin D [4]. Moreover, previous findings indicated that deficiency of 25-hydroxyvitamin D significantly elevates, slightly decreases, and obviously reduced SLE risk in nineteen different case control studies. However, these epidemiological studies did not explore whether vitamin D deficiency is the cause or the effect [29] In this bidirectional MR study, the results showed that determined SLE was causally associated with decreased VD levels, indicating that vitamin D deficiency is climate related to SLE.

The casual effect of SLE on vitamin D levels has several potential explanations. According to a large number of clinical experience, vitamin D deficiency is very common in SLE patients. Analysis shows that this is related to race, geography, and season-related factors, but the influence level of various factors is not very clear [30]. Furthermore, vitamin D is reported to be a negative acute phase reactant for SLE, implying that its levels decrease in acute inflammatory conditions [29]. A previous study reported a modulatory role of VD on immune system [31]. Some clinical trials showed that vitamin D in patients with SLE [32] suppressed the disease activity, fatigue, and risk of thrombosis. Elsewhere, it was found that vitamin D supplementation reduced proteinuria, increased complement levels, and improved the global disease activity in SLE [2].

This study has several strengths. To begin with, the MR approach used allowed causal inference free from confounding and reverse causation and minimized potential biases based on relative core assumptions [33]. Furthermore, the casual effect was explored using two models (extracted SNPs with confounders or not) based on various large-scale consortium data which increases the statistical power [8]. Moreover, in this study process, sensitivity analysis was conducted, and the results showed the consistent effects between vitamin D and SLE. In conclusion, the genetic variants were located; therefore, the gene-gene interaction had weak relation with estimate result [34].

There are few shortcomings in the current study. First, potential nonlinear association of VD and SLE risk was not evaluated due to lack of individual data. Second, the contribution of VD related SNPs to the variation ratio is not high, so the correlation between VD concentration and SLE cannot be well explained in the analysis process. Therefore, statistical analysis of other variables is needed. However, *F*-statistic above 10 minimizes any bias caused by weak instruments. Finally, the study population only included European ancestry. Studies report that the casual effect between vitamin D and SLE is inconsistent across races. This implies that the results cannot be extended to non-European populations. More studies should be conducted in other ethnicities.

## 5. Conclusion

These findings indicated that genetically determined SLE had a negative effect on the vitamin D and 25-hydroxyvitamin D levels. Future studies, including random controlled clinical trials, should further evaluate the association and potential role between serum VD levels and SLE.

## Figures and Tables

**Table 1:** Details on GWAS predictive strength of IVs used in Mendelian randomization analyses.

Study design		Details of GWAS (exposures)		Strength of IVs			
Exposure	Outcome	Consortium	Sample size	Model	No. of SNPs	*R* ^2^	*F*-statistic
Vitamin D	Systemic lupus erythematosus	UK biobank	304,818	Model 1	53	0.585	33.863
				Model 2	46	0.537	30.883

25-Hydroxyvitamin D	Systemic lupus erythematosus	UK biobank	401,460	Model 1	160	0.459	12.452
				Model 2	147	0.431	11.827

Systemic lupus erythematosus	Vitamin D	FinnGen biobank	213,683	Model 1	8	0.088	23.433
				Model 2	7	0.071	21.837

Systemic lupus erythematosus	25-Hydroxyvitamin D	FinnGen biobank	213,683	Model 1	38	0.551	31.152
				Model 2	33	0.463	26.169

Model 1: SNPs associated with any potential confounders were not extracted. Model 2: SNPs associated with any potential confounders were extracted. SNP: single-nucleotide polymorphisms.

**Table 2:** MR-PRESSO estimates between vitamin D and systemic lupus erythematosus.

Exposure	Outcome	Model	Raw estimates				Outlier corrected estimates				Distortion test
			nSNP	Beta	OR (95% CI)	*P* value	nSNP	Beta	OR (95% CI)	*P* value	*P* value
SLE	Vitamin D	Model 1	8	-0.008	0.992 (0.986, 0.998)	0.047	8	NA	NA	NA	NA
SLE	Vitamin D	Model 2	7	-0.009	0.991 (0.985, 0.997)	0.041	7	NA	NA	NA	NA
SLE	25-Hydroxyvitamin D	Model 1	38	-0.004	0.996 (0.992, 0.999)	0.04	36	-0.004	0.996 (0.992, 0.999)	0.008	0.747
SLE	25-Hydroxyvitamin D	Model 2	33	-0.005	0.995 (0.991, 0.999)	0.014	32	-0.006	0.994 (0.990, 0.998)	0.004	0.772
Vitamin D	SLE	Model 1	53	-0.303	0.739 (0.419, 1.301)	0.299	53	NA	NA	NA	NA
Vitamin D	SLE	Model 2	46	-0.27	0.763 (0.442, 1.319)	0.337	46	NA	NA	NA	NA
25-Hydroxyvitamin D	SLE	Model 1	160	0.432	1.540 (0.811, 2.924)	0.188	160	NA	NA	NA	NA
25-Hydroxyvitamin D	SLE	Model 2	147	0.483	1.621 (0.839, 3.132)	0.153	147	NA	NA	NA	NA

SLE: systemic lupus erythematosus; OR: odds ratio; SNP: single-nucleotide polymorphisms.

## Data Availability

Data are presented in the supplementary materials.

## Supplementary Materials

Supplementary Materials Supplementary table 1: harmonized dataset of univariate Mendelian randomization for the effect of SLE on vitamin D with model 1. Supplementary table 2: harmonized dataset of univariate Mendelian randomization for the effect of SLE on vitamin D with model 2. Supplementary table 3: harmonized dataset of univariate Mendelian randomization for the effect of SLE on 25-hydroxyvitamin D with model 1. Supplementary table 4: harmonized dataset of univariate Mendelian randomization for the effect of SLE on 25-hydroxyvitamin D with model 2. Supplementary table 5: harmonized dataset of univariate Mendelian randomization for the effect of vitamin D on SLE with model 1. Supplementary table 6: harmonized dataset of univariate Mendelian randomization for the effect of vitamin D on SLE with model 2. Supplementary table 7: harmonized dataset of univariate Mendelian randomization for the effect of 25-hydroxyvitamin D on SLE with model 1. Supplementary table 8: harmonized dataset of univariate Mendelian randomization for the effect of 25-hydroxyvitamin D on SLE with model 2. Supplementary table 9: MR-PRESSO estimates between vitamin D and systemic lupus erythematosus. Supplementary figure 1: IVW radial for the effect of SLE on vitamin D with model 1. Supplementary figure 2: IVW radial for the effect of SLE on vitamin D with model 2. Supplementary figure 3: diagnostic plots generated by MR-RAPS of SLE on vitamin D with model 1. Supplementary figure 4: diagnostic plots generated by MR-RAPS of SLE on vitamin D with model 2. Supplementary figure 5: IVW radial for the effect of SLE on 25-hydroxyvitamin D levels with model 1. Supplementary figure 6: IVW radial for the effect of SLE on 25-hydroxyvitamin D levels with model 2. Supplementary figure 7: IVW radial for the effect of vitamin D on SLE with model 1. Supplementary figure 8: IVW radial for the effect of vitamin D on SLE with model 2. Supplementary figure 9: diagnostic plots generated by MR-RAPS of vitamin D on SLE with model 1. Supplementary figure 10: diagnostic plots generated by MR-RAPS of vitamin D on SLE with model 2. Supplementary figure 11: IVW radial for the effect of 25-hydroxyvitamin D levels on SLE with model 1. Supplementary figure 12: IVW radial for the effect of 25-hydroxyvitamin D levels on SLE with model 2. Supplementary figure 13: diagnostic plots generated by MR-RAPS of 25-hydroxyvitamin D levels on SLE with model 1. Supplementary figure 14: diagnostic plots generated by MR-RAPS of 25-hydroxyvitamin D levels on SLE with model 2.
