# Supplementary material for: Is Vitamin D Deficiency the Cause or the Effect of Systemic Lupus Erythematosus: Evidence from Bidirectional Mendelian Randomization Analysis
Source: J Immunol Res. 2022 Sep 21;2022:8689777. doi: 10.1155/2022/8689777 (PMC9519279; doi:10.1155/2022/8689777)
Supplement: Supplementary Materials — Supplementary table 1: harmonized dataset of univariate Mendelian randomization for the effect of SLE on vitamin D with model 1. Supplementary table 2: harmonized dataset of univariate Mendelian randomization for the effect of SLE on vitamin D with model 2. Supplementary table 3: harmonized dataset of univariate Mendelian randomization for the effect of SLE on 25-hydroxyvitamin D with model 1. Supplementary table 4: harmonized dataset of univariate Mendelian randomization for the effect of SLE on 25-hydroxyvitamin D with model 2. Supplementary table 5: harmonized dataset of univariate Mendelian randomization for the effect of vitamin D on SLE with model 1. Supplementary table 6: harmonized dataset of univariate Mendelian randomization for the effect of vitamin D on SLE with model 2. Supplementary table 7: harmonized dataset of univariate Mendelian randomization for the effect of 25-hydroxyvitamin D on SLE with model 1. Supplementary table 8: harmonized dataset of univariate Mendelian randomization for the effect of 25-hydroxyvitamin D on SLE with model 2. Supplementary table 9: MR-PRESSO estimates between vitamin D and systemic lupus erythematosus. Supplementary figure 1: IVW radial for the effect of SLE on vitamin D with model 1. Supplementary figure 2: IVW radial for the effect of SLE on vitamin D with model 2. Supplementary figure 3: diagnostic plots generated by MR-RAPS of SLE on vitamin D with model 1. Supplementary figure 4: diagnostic plots generated by MR-RAPS of SLE on vitamin D with model 2. Supplementary figure 5: IVW radial for the effect of SLE on 25-hydroxyvitamin D levels with model 1. Supplementary figure 6: IVW radial for the effect of SLE on 25-hydroxyvitamin D levels with model 2. Supplementary figure 7: IVW radial for the effect of vitamin D on SLE with model 1. Supplementary figure 8: IVW radial for the effect of vitamin D on SLE with model 2. Supplementary figure 9: diagnostic plots generated by MR-RAPS of vitamin D on SLE with model 1. Supplem [file 8689777.f1.zip › Supplymentary tables.docx]

| Supplementary table 1. Harmonized dataset of univariate Mendelian randomization for the effect of SLE on vitamin D with model 1 | | | | | | | | | | | |
| --- | --- | --- | --- | --- | --- | --- | --- | --- | --- | --- | --- |
|  |  |  |  |  |  | Exposure | | | Outcome | | |
| SNP | effect_allele.exposure | other_allele.exposure | effect_allele.outcome | other_allele.outcome | Chromosome | Effect | SE | P-value | Effect | SE | P-value |
| rs12524498 | T | G | T | G | 6 | -0.673345 | 0.120793 | 2.48E-08 | 0.0116 | 0.0087 | 0.1862 |
| rs13332649 | G | A | G | A | 16 | -0.314711 | 0.0375683 | 5.43E-17 | 0.0044 | 0.0029 | 0.1324 |
| rs17849501 | T | C | T | C | 1 | 0.81093 | 0.0498642 | 1.81E-59 | -0.0058 | 0.0055 | 0.2961 |
| rs2431697 | C | T | C | T | 5 | -0.223144 | 0.0292964 | 2.60E-14 | -0.0026 | 0.0025 | 0.2864 |
| rs389884 | G | A | G | A | 6 | 0.928219 | 0.0432319 | 2.92E-102 | -0.0083 | 0.0036 | 0.02322 |
| rs6679677 | A | C | A | C | 1 | 0.336472 | 0.0464854 | 4.55E-13 | 0.0036 | 0.0041 | 0.374 |
| rs7097397 | A | G | A | G | 10 | -0.18633 | 0.0287118 | 8.60E-11 | -2.00E-04 | 0.0025 | 0.9367 |
| rs73068668 | A | G | A | G | 19 | -0.314711 | 0.0574903 | 4.40E-08 | 0.0122 | 0.0049 | 0.01295 |

| Supplementary table 2. Harmonized dataset of univariate Mendelian randomization for the effect of SLE on vitamin D with model 2 | | | | | | | | | | | |
| --- | --- | --- | --- | --- | --- | --- | --- | --- | --- | --- | --- |
|  |  |  |  |  |  | Exposure | | | Outcome | | |
| SNP | effect_allele.exposure | other_allele.exposure | effect_allele.outcome | other_allele.outcome | Chromosome | Effect | SE | P-value | Effect | SE | P-value |
| rs12524498 | T | G | T | G | 6 | -0.673345 | 0.120793 | 2.48E-08 | 0.0116 | 0.0087 | 0.1862 |
| rs13332649 | G | A | G | A | 16 | -0.314711 | 0.0375683 | 5.43E-17 | 0.0044 | 0.0029 | 0.1324 |
| rs17849501 | T | C | T | C | 1 | 0.81093 | 0.0498642 | 1.81E-59 | -0.0058 | 0.0055 | 0.2961 |
| rs2431697 | C | T | C | T | 5 | -0.223144 | 0.0292964 | 2.60E-14 | -0.0026 | 0.0025 | 0.2864 |
| rs389884 | G | A | G | A | 6 | 0.928219 | 0.0432319 | 2.92E-102 | -0.0083 | 0.0036 | 0.02322 |
| rs7097397 | A | G | A | G | 10 | -0.18633 | 0.0287118 | 8.60E-11 | -2.00E-04 | 0.0025 | 0.9367 |
| rs73068668 | A | G | A | G | 19 | -0.314711 | 0.0574903 | 4.40E-08 | 0.0122 | 0.0049 | 0.01295 |

| Supplementary table 3. Harmonized dataset of univariate Mendelian randomization for the effect of SLE on 25-hydroxyvitamin D with model 1 | | | | | | | | | | | |
| --- | --- | --- | --- | --- | --- | --- | --- | --- | --- | --- | --- |
|  |  |  |  |  |  | Exposure | | | Outcome | | |
| SNP | effect_allele.exposure | other_allele.exposure | effect_allele.outcome | other_allele.outcome | Chromosome | Effect | SE | P-value | Effect | SE | P-value |
| rs10048743 | T | G | T | G | 2 | -0.231112 | 0.0412056 | 2.04E-08 | -0.007983 | 0.00281 | 0.00452 |
| rs10200680 | T | C | T | C | 2 | -0.248461 | 0.0424835 | 4.96E-09 | 0.003555 | 0.002751 | 0.19625 |
| rs1078324 | A | C | A | C | 5 | -0.71335 | 0.0781665 | 7.11E-20 | 0.004967 | 0.004221 | 0.239214 |
| rs10912578 | G | A | G | A | 1 | -0.24686 | 0.0309918 | 1.65E-15 | -0.002294 | 0.00209 | 0.272451 |
| rs1143679 | A | G | A | G | 16 | 0.582216 | 0.0399866 | 5.03E-48 | -0.009747 | 0.003246 | 0.002684 |
| rs12094036 | C | T | C | T | 1 | -0.328504 | 0.0578595 | 1.37E-08 | -0.000625 | 0.003593 | 0.861963 |
| rs12524498 | T | G | T | G | 6 | -0.673345 | 0.120793 | 2.48E-08 | 0.007281 | 0.007189 | 0.311164 |
| rs13019891 | T | G | T | G | 2 | -0.562119 | 0.0290336 | 1.65E-83 | -0.003079 | 0.00196 | 0.116247 |
| rs13136219 | T | C | T | C | 4 | -0.174353 | 0.027787 | 3.50E-10 | 0.002132 | 0.001999 | 0.286214 |
| rs13332649 | G | A | G | A | 16 | -0.314711 | 0.0375683 | 5.43E-17 | 0.00388 | 0.002333 | 0.0963053 |
| rs1464446 | T | G | T | G | 3 | -0.328504 | 0.0401497 | 2.79E-16 | 0.003596 | 0.002468 | 0.145065 |
| rs17849501 | T | C | T | C | 1 | 0.81093 | 0.0498642 | 1.81E-59 | -0.001513 | 0.004395 | 0.730683 |
| rs2459611 | T | C | T | C | 2 | 0.261365 | 0.045245 | 7.62E-09 | -0.001351 | 0.003336 | 0.685558 |
| rs268124 | T | C | T | C | 2 | 0.18633 | 0.0323703 | 8.60E-09 | -0.005258 | 0.002197 | 0.016739 |
| rs28361029 | A | G | A | G | 6 | -0.385662 | 0.0613604 | 3.27E-10 | 0.025671 | 0.020988 | 0.221261 |
| rs34703115 | C | T | C | T | 2 | -0.616186 | 0.104778 | 4.08E-09 | 0.006468 | 0.006178 | 0.29509 |
| rs35000415 | T | C | T | C | 7 | 0.587787 | 0.041539 | 1.86E-45 | -0.008222 | 0.003084 | 0.007694 |
| rs35251378 | A | G | A | G | 19 | -0.235722 | 0.0324266 | 3.61E-13 | 0.004389 | 0.002144 | 0.040709 |
| rs353608 | G | A | G | A | 11 | 0.18633 | 0.0280198 | 2.93E-11 | -0.000631 | 0.001939 | 0.744957 |
| rs389884 | G | A | G | A | 6 | 0.928219 | 0.0432319 | 2.92E-102 | -0.007982 | 0.002897 | 0.005887 |
| rs4274624 | T | C | T | C | 2 | -0.559616 | 0.0326791 | 9.73E-66 | -0.003633 | 0.002326 | 0.11829 |
| rs4388254 | T | C | T | C | 5 | 0.378436 | 0.0603977 | 3.71E-10 | -0.003351 | 0.005163 | 0.516366 |
| rs4661543 | G | T | G | T | 1 | 0.274437 | 0.0423755 | 9.40E-11 | 0.000668 | 0.002938 | 0.820122 |
| rs4916215 | T | C | T | C | 1 | 0.223144 | 0.0339693 | 5.07E-11 | -0.000933 | 0.002183 | 0.669095 |
| rs58688157 | G | A | G | A | 11 | -0.223144 | 0.0335647 | 2.97E-11 | -0.004906 | 0.002168 | 0.023651 |
| rs58721818 | T | C | T | C | 6 | 0.65752 | 0.0755941 | 3.38E-18 | -0.004562 | 0.005578 | 0.413428 |
| rs597808 | G | A | G | A | 12 | -0.162519 | 0.0294736 | 3.51E-08 | -0.002745 | 0.001954 | 0.16009 |
| rs6671847 | A | G | A | G | 1 | 0.198851 | 0.0289651 | 6.64E-12 | 0.005307 | 0.001945 | 0.006385 |
| rs6679677 | A | C | A | C | 1 | 0.336472 | 0.0464854 | 4.55E-13 | 0.003562 | 0.003201 | 0.265819 |
| rs6889239 | C | T | C | T | 5 | 0.277632 | 0.03174 | 2.19E-18 | -0.001674 | 0.00229 | 0.464665 |
| rs7097397 | A | G | A | G | 10 | -0.18633 | 0.0287118 | 8.60E-11 | 0.000879 | 0.002017 | 0.662966 |
| rs73050535 | T | C | T | C | 12 | -0.71335 | 0.124134 | 9.11E-09 | 0.007186 | 0.006415 | 0.262632 |
| rs73068668 | A | G | A | G | 19 | -0.314711 | 0.0574903 | 4.40E-08 | 0.009879 | 0.0037 | 0.0076061 |
| rs7768653 | T | C | T | C | 6 | -0.207014 | 0.0296891 | 3.11E-12 | 0.00084 | 0.001985 | 0.672174 |
| rs7823055 | T | G | T | G | 8 | -0.350657 | 0.0286208 | 1.64E-34 | -0.000517 | 0.00198 | 0.793853 |
| rs7899626 | T | C | T | C | 10 | 0.182322 | 0.0332532 | 4.19E-08 | -0.001148 | 0.002145 | 0.592656 |
| rs9274357 | T | C | T | C | 6 | 0.457425 | 0.0351961 | 1.28E-38 | -0.026379 | 0.021112 | 0.21147 |
| rs9852014 | G | A | G | A | 3 | 0.620577 | 0.0492727 | 2.26E-36 | 0.003447 | 0.003841 | 0.369471 |

| Supplementary table 4. Harmonized dataset of univariate Mendelian randomization for the effect of SLE on 25-hydroxyvitamin D with model 2 | | | | | | | | | | | |
| --- | --- | --- | --- | --- | --- | --- | --- | --- | --- | --- | --- |
|  |  |  |  |  |  | Exposure | | | Outcome | | |
| SNP | effect_allele.exposure | other_allele.exposure | effect_allele.outcome | other_allele.outcome | Chromosome | Effect | SE | P-value | Effect | SE | P-value |
| rs10200680 | T | C | T | C | 2 | -0.248461 | 0.0424835 | 4.96E-09 | 0.003555 | 0.002751 | 0.19625 |
| rs1078324 | A | C | A | C | 5 | -0.71335 | 0.0781665 | 7.11E-20 | 0.004967 | 0.004221 | 0.239214 |
| rs10912578 | G | A | G | A | 1 | -0.24686 | 0.0309918 | 1.65E-15 | -0.002294 | 0.00209 | 0.272451 |
| rs1143679 | A | G | A | G | 16 | 0.582216 | 0.0399866 | 5.03E-48 | -0.009747 | 0.003246 | 0.002684 |
| rs12094036 | C | T | C | T | 1 | -0.328504 | 0.0578595 | 1.37E-08 | -0.000625 | 0.003593 | 0.861963 |
| rs12524498 | T | G | T | G | 6 | -0.673345 | 0.120793 | 2.48E-08 | 0.007281 | 0.007189 | 0.311164 |
| rs13136219 | T | C | T | C | 4 | -0.174353 | 0.027787 | 3.50E-10 | 0.002132 | 0.001999 | 0.286214 |
| rs13332649 | G | A | G | A | 16 | -0.314711 | 0.0375683 | 5.43E-17 | 0.00388 | 0.002333 | 0.0963053 |
| rs1464446 | T | G | T | G | 3 | -0.328504 | 0.0401497 | 2.79E-16 | 0.003596 | 0.002468 | 0.145065 |
| rs17849501 | T | C | T | C | 1 | 0.81093 | 0.0498642 | 1.81E-59 | -0.001513 | 0.004395 | 0.730683 |
| rs2459611 | T | C | T | C | 2 | 0.261365 | 0.045245 | 7.62E-09 | -0.001351 | 0.003336 | 0.685558 |
| rs268124 | T | C | T | C | 2 | 0.18633 | 0.0323703 | 8.60E-09 | -0.005258 | 0.002197 | 0.016739 |
| rs28361029 | A | G | A | G | 6 | -0.385662 | 0.0613604 | 3.27E-10 | 0.025671 | 0.020988 | 0.221261 |
| rs34703115 | C | T | C | T | 2 | -0.616186 | 0.104778 | 4.08E-09 | 0.006468 | 0.006178 | 0.29509 |
| rs35000415 | T | C | T | C | 7 | 0.587787 | 0.041539 | 1.86E-45 | -0.008222 | 0.003084 | 0.007694 |
| rs35251378 | A | G | A | G | 19 | -0.235722 | 0.0324266 | 3.61E-13 | 0.004389 | 0.002144 | 0.040709 |
| rs353608 | G | A | G | A | 11 | 0.18633 | 0.0280198 | 2.93E-11 | -0.000631 | 0.001939 | 0.744957 |
| rs4274624 | T | C | T | C | 2 | -0.559616 | 0.0326791 | 9.73E-66 | -0.003633 | 0.002326 | 0.11829 |
| rs4388254 | T | C | T | C | 5 | 0.378436 | 0.0603977 | 3.71E-10 | -0.003351 | 0.005163 | 0.516366 |
| rs4661543 | G | T | G | T | 1 | 0.274437 | 0.0423755 | 9.40E-11 | 0.000668 | 0.002938 | 0.820122 |
| rs4916215 | T | C | T | C | 1 | 0.223144 | 0.0339693 | 5.07E-11 | -0.000933 | 0.002183 | 0.669095 |
| rs58688157 | G | A | G | A | 11 | -0.223144 | 0.0335647 | 2.97E-11 | -0.004906 | 0.002168 | 0.023651 |
| rs58721818 | T | C | T | C | 6 | 0.65752 | 0.0755941 | 3.38E-18 | -0.004562 | 0.005578 | 0.413428 |
| rs6671847 | A | G | A | G | 1 | 0.198851 | 0.0289651 | 6.64E-12 | 0.005307 | 0.001945 | 0.006385 |
| rs6889239 | C | T | C | T | 5 | 0.277632 | 0.03174 | 2.19E-18 | -0.001674 | 0.00229 | 0.464665 |
| rs7097397 | A | G | A | G | 10 | -0.18633 | 0.0287118 | 8.60E-11 | 0.000879 | 0.002017 | 0.662966 |
| rs73050535 | T | C | T | C | 12 | -0.71335 | 0.124134 | 9.11E-09 | 0.007186 | 0.006415 | 0.262632 |
| rs73068668 | A | G | A | G | 19 | -0.314711 | 0.0574903 | 4.40E-08 | 0.009879 | 0.0037 | 0.0076061 |
| rs7768653 | T | C | T | C | 6 | -0.207014 | 0.0296891 | 3.11E-12 | 0.00084 | 0.001985 | 0.672174 |
| rs7823055 | T | G | T | G | 8 | -0.350657 | 0.0286208 | 1.64E-34 | -0.000517 | 0.00198 | 0.793853 |
| rs7899626 | T | C | T | C | 10 | 0.182322 | 0.0332532 | 4.19E-08 | -0.001148 | 0.002145 | 0.592656 |
| rs9274357 | T | C | T | C | 6 | 0.457425 | 0.0351961 | 1.28E-38 | -0.026379 | 0.021112 | 0.21147 |
| rs9852014 | G | A | G | A | 3 | 0.620577 | 0.0492727 | 2.26E-36 | 0.003447 | 0.003841 | 0.369471 |

| Supplementary table 5. Harmonized dataset of univariate Mendelian randomization for the effect of vitamin D on SLE with model 1 | | | | | | | | | | | |
| --- | --- | --- | --- | --- | --- | --- | --- | --- | --- | --- | --- |
|  |  |  |  |  |  | Exposure | | | Outcome | | |
| SNP | effect_allele.exposure | other_allele.exposure | effect_allele.outcome | other_allele.outcome | Chromosome | Effect | SE | P-value | Effect | SE | P-value |
| rs10426201 | A | G | A | G | 19 | -0.067 | 0.0033 | 1.36E-92 | 0.1476 | 0.0806 | 0.0670394 |
| rs1047891 | A | C | A | C | 2 | -0.0158 | 0.0026 | 1.67E-09 | 0.0245 | 0.0658 | 0.709999 |
| rs10489615 | G | A | G | A | 1 | 0.0137 | 0.0025 | 4.22E-08 | -0.0291 | 0.0617 | 0.636999 |
| rs10859995 | C | T | C | T | 12 | -0.0451 | 0.0025 | 4.00E-74 | 0.0471 | 0.0646 | 0.4662 |
| rs11023463 | C | A | C | A | 11 | -0.0187 | 0.0034 | 2.68E-08 | -0.069 | 0.0681 | 0.3104 |
| rs11205009 | C | T | C | T | 1 | 0.0382 | 0.0043 | 3.61E-19 | 0.1217 | 0.1309 | 0.3528 |
| rs113137180 | G | T | G | T | 11 | -0.064 | 0.0056 | 2.15E-30 | 0.1135 | 0.1205 | 0.3464 |
| rs115280883 | T | C | T | C | 4 | -0.0965 | 0.0124 | 8.38E-15 | -0.0905 | 0.3653 | 0.8044 |
| rs11542462 | A | G | A | G | 16 | -0.0277 | 0.0036 | 1.00E-14 | 0.1001 | 0.1048 | 0.3397 |
| rs116924445 | T | C | T | C | 16 | 0.0528 | 0.0096 | 3.22E-08 | -0.0833 | 0.3942 | 0.8327 |
| rs11732896 | A | G | A | G | 4 | -0.0176 | 0.0027 | 3.77E-11 | -0.0889 | 0.07 | 0.204 |
| rs12123821 | T | C | T | C | 1 | 0.0827 | 0.0059 | 2.46E-44 | -0.1641 | 0.1591 | 0.3024 |
| rs1229984 | C | T | C | T | 4 | -0.0469 | 0.008 | 3.62E-09 | -0.1188 | 0.4048 | 0.7692 |
| rs1260326 | C | T | C | T | 2 | 0.02 | 0.0025 | 1.41E-15 | 0.0528 | 0.0644 | 0.4124 |
| rs12740374 | T | G | T | G | 1 | 0.0218 | 0.0029 | 6.62E-14 | 0.0082 | 0.0746 | 0.9129 |
| rs13330068 | T | G | T | G | 16 | 0.0191 | 0.0035 | 3.54E-08 | -0.0871 | 0.1043 | 0.4035 |
| rs1532085 | G | A | G | A | 15 | 0.0254 | 0.0025 | 2.95E-24 | -0.0199 | 0.0618 | 0.747599 |
| rs157580 | A | G | A | G | 19 | -0.0157 | 0.0025 | 2.91E-10 | -0.0084 | 0.0678 | 0.9011 |
| rs16961689 | C | A | C | A | 18 | -0.0265 | 0.0048 | 2.90E-08 | 0.0845 | 0.1131 | 0.4547 |
| rs17217119 | G | A | G | A | 20 | -0.0395 | 0.0031 | 2.91E-38 | -0.0343 | 0.0724 | 0.6356 |
| rs1792277 | T | C | T | C | 11 | -0.1016 | 0.0031 | 1.00E-200 | -0.0387 | 0.0642 | 0.547 |
| rs1800588 | T | C | T | C | 15 | -0.0336 | 0.0029 | 3.73E-30 | -0.0376 | 0.0712 | 0.597601 |
| rs1820073 | C | T | C | T | 5 | 0.0141 | 0.0025 | 1.23E-08 | -0.0715 | 0.0631 | 0.2576 |
| rs2131925 | T | G | T | G | 1 | -0.0225 | 0.0025 | 1.21E-18 | 0.1086 | 0.0694 | 0.1173 |
| rs2205262 | A | C | A | C | 8 | -0.0225 | 0.0025 | 7.47E-20 | 0.0128 | 0.0613 | 0.8349 |
| rs2207132 | A | G | A | G | 20 | -0.0399 | 0.0069 | 8.15E-09 | 0.0709 | 0.1238 | 0.566601 |
| rs2246012 | C | T | C | T | 6 | -0.0236 | 0.0032 | 3.28E-13 | 0.062 | 0.0724 | 0.3915 |
| rs2539986 | T | C | T | C | 2 | -0.0187 | 0.003 | 5.94E-10 | -0.1676 | 0.0703 | 0.0171002 |
| rs2642442 | T | C | T | C | 1 | -0.0189 | 0.0026 | 5.22E-13 | -0.0158 | 0.0674 | 0.8151 |
| rs2847500 | A | G | A | G | 11 | -0.0207 | 0.0037 | 1.66E-08 | 0.086 | 0.0874 | 0.3249 |
| rs34762726 | A | G | A | G | 3 | -0.0172 | 0.0027 | 1.53E-10 | 0.1226 | 0.0626 | 0.0503605 |
| rs34937961 | T | C | T | C | 15 | -0.0152 | 0.0028 | 4.07E-08 | -0.0983 | 0.0664 | 0.1387 |
| rs35635959 | C | T | C | T | 17 | -0.0164 | 0.0027 | 1.02E-09 | -0.1314 | 0.071 | 0.0642999 |
| rs4149056 | C | T | C | T | 12 | -0.0245 | 0.0034 | 8.51E-13 | 0.143 | 0.0769 | 0.0629695 |
| rs4266261 | C | T | C | T | 4 | 0.0503 | 0.0039 | 1.17E-38 | 0.0185 | 0.0905 | 0.8377 |
| rs4920359 | G | A | G | A | 1 | -0.0214 | 0.0025 | 3.10E-17 | -0.1217 | 0.0636 | 0.0557596 |
| rs55791371 | C | A | C | A | 19 | 0.0277 | 0.0038 | 1.80E-13 | -0.099 | 0.1013 | 0.3284 |
| rs56219219 | T | C | T | C | 4 | 0.0221 | 0.0037 | 1.65E-09 | -0.091 | 0.1093 | 0.4048 |
| rs56675301 | C | T | C | T | 1 | 0.0181 | 0.0028 | 4.50E-11 | -0.0379 | 0.0681 | 0.5777 |
| rs58542926 | T | C | T | C | 19 | 0.0448 | 0.0046 | 1.94E-22 | 0.0278 | 0.1234 | 0.8217 |
| rs62302166 | G | T | G | T | 4 | -0.162 | 0.0051 | 1.00E-200 | 0.0139 | 0.1348 | 0.9179 |
| rs6576006 | C | A | C | A | 14 | 0.0158 | 0.0026 | 1.06E-09 | -0.0313 | 0.0629 | 0.6191 |
| rs6600937 | A | G | A | G | 4 | 0.0208 | 0.0027 | 9.06E-15 | 0.0541 | 0.0624 | 0.386 |
| rs6754295 | G | T | G | T | 2 | 0.0162 | 0.0029 | 2.09E-08 | 0.0336 | 0.0683 | 0.6231 |
| rs6814839 | G | A | G | A | 4 | -0.1166 | 0.0048 | 7.73E-131 | 0.03 | 0.1122 | 0.7891 |
| rs71473824 | T | C | T | C | 11 | -0.0552 | 0.0068 | 3.68E-16 | -0.2885 | 0.1567 | 0.0655904 |
| rs7499892 | T | C | T | C | 16 | 0.0244 | 0.0031 | 8.50E-15 | 0.0487 | 0.0806 | 0.5456 |
| rs7652808 | G | T | G | T | 3 | -0.0171 | 0.0026 | 2.34E-11 | 0.0645 | 0.0686 | 0.3476 |
| rs79444700 | C | T | C | T | 4 | 0.0851 | 0.006 | 6.70E-46 | -0.0316 | 0.1708 | 0.8533 |
| rs804280 | A | C | A | C | 8 | 0.0146 | 0.0025 | 6.27E-09 | -0.0653 | 0.09 | 0.4678 |
| rs8123293 | G | A | G | A | 20 | 0.0275 | 0.0038 | 4.41E-13 | 0.0859 | 0.0793 | 0.2791 |
| rs883541 | A | G | A | G | 17 | 0.0173 | 0.0029 | 2.61E-09 | -0.1027 | 0.0718 | 0.1527 |
| rs9469112 | T | C | T | C | 6 | -0.0184 | 0.0034 | 4.84E-08 | 0.2507 | 0.1194 | 0.0357899 |

| Supplementary table 6. Harmonized dataset of univariate Mendelian randomization for the effect of vitamin D on SLE with model 2 | | | | | | | | | | | |
| --- | --- | --- | --- | --- | --- | --- | --- | --- | --- | --- | --- |
|  |  |  |  |  |  | Exposure | | | Outcome | | |
| SNP | effect_allele.exposure | other_allele.exposure | effect_allele.outcome | other_allele.outcome | Chromosome | Effect | SE | P-value | Effect | SE | P-value |
| rs10426201 | A | G | A | G | 19 | -0.067 | 0.0033 | 1.36E-92 | 0.1476 | 0.0806 | 0.0670394 |
| rs1047891 | A | C | A | C | 2 | -0.0158 | 0.0026 | 1.67E-09 | 0.0245 | 0.0658 | 0.709999 |
| rs10489615 | G | A | G | A | 1 | 0.0137 | 0.0025 | 4.22E-08 | -0.0291 | 0.0617 | 0.636999 |
| rs10859995 | C | T | C | T | 12 | -0.0451 | 0.0025 | 4.00E-74 | 0.0471 | 0.0646 | 0.4662 |
| rs11023463 | C | A | C | A | 11 | -0.0187 | 0.0034 | 2.68E-08 | -0.069 | 0.0681 | 0.3104 |
| rs11205009 | C | T | C | T | 1 | 0.0382 | 0.0043 | 3.61E-19 | 0.1217 | 0.1309 | 0.3528 |
| rs113137180 | G | T | G | T | 11 | -0.064 | 0.0056 | 2.15E-30 | 0.1135 | 0.1205 | 0.3464 |
| rs115280883 | T | C | T | C | 4 | -0.0965 | 0.0124 | 8.38E-15 | -0.0905 | 0.3653 | 0.8044 |
| rs11542462 | A | G | A | G | 16 | -0.0277 | 0.0036 | 1.00E-14 | 0.1001 | 0.1048 | 0.3397 |
| rs116924445 | T | C | T | C | 16 | 0.0528 | 0.0096 | 3.22E-08 | -0.0833 | 0.3942 | 0.8327 |
| rs11732896 | A | G | A | G | 4 | -0.0176 | 0.0027 | 3.77E-11 | -0.0889 | 0.07 | 0.204 |
| rs12123821 | T | C | T | C | 1 | 0.0827 | 0.0059 | 2.46E-44 | -0.1641 | 0.1591 | 0.3024 |
| rs12740374 | T | G | T | G | 1 | 0.0218 | 0.0029 | 6.62E-14 | 0.0082 | 0.0746 | 0.9129 |
| rs13330068 | T | G | T | G | 16 | 0.0191 | 0.0035 | 3.54E-08 | -0.0871 | 0.1043 | 0.4035 |
| rs1532085 | G | A | G | A | 15 | 0.0254 | 0.0025 | 2.95E-24 | -0.0199 | 0.0618 | 0.747599 |
| rs157580 | A | G | A | G | 19 | -0.0157 | 0.0025 | 2.91E-10 | -0.0084 | 0.0678 | 0.9011 |
| rs16961689 | C | A | C | A | 18 | -0.0265 | 0.0048 | 2.90E-08 | 0.0845 | 0.1131 | 0.4547 |
| rs17217119 | G | A | G | A | 20 | -0.0395 | 0.0031 | 2.91E-38 | -0.0343 | 0.0724 | 0.6356 |
| rs1792277 | T | C | T | C | 11 | -0.1016 | 0.0031 | 1.00E-200 | -0.0387 | 0.0642 | 0.547 |
| rs1800588 | T | C | T | C | 15 | -0.0336 | 0.0029 | 3.73E-30 | -0.0376 | 0.0712 | 0.597601 |
| rs1820073 | C | T | C | T | 5 | 0.0141 | 0.0025 | 1.23E-08 | -0.0715 | 0.0631 | 0.2576 |
| rs2131925 | T | G | T | G | 1 | -0.0225 | 0.0025 | 1.21E-18 | 0.1086 | 0.0694 | 0.1173 |
| rs2205262 | A | C | A | C | 8 | -0.0225 | 0.0025 | 7.47E-20 | 0.0128 | 0.0613 | 0.8349 |
| rs2207132 | A | G | A | G | 20 | -0.0399 | 0.0069 | 8.15E-09 | 0.0709 | 0.1238 | 0.566601 |
| rs2539986 | T | C | T | C | 2 | -0.0187 | 0.003 | 5.94E-10 | -0.1676 | 0.0703 | 0.0171002 |
| rs2642442 | T | C | T | C | 1 | -0.0189 | 0.0026 | 5.22E-13 | -0.0158 | 0.0674 | 0.8151 |
| rs2847500 | A | G | A | G | 11 | -0.0207 | 0.0037 | 1.66E-08 | 0.086 | 0.0874 | 0.3249 |
| rs34937961 | T | C | T | C | 15 | -0.0152 | 0.0028 | 4.07E-08 | -0.0983 | 0.0664 | 0.1387 |
| rs4149056 | C | T | C | T | 12 | -0.0245 | 0.0034 | 8.51E-13 | 0.143 | 0.0769 | 0.0629695 |
| rs4266261 | C | T | C | T | 4 | 0.0503 | 0.0039 | 1.17E-38 | 0.0185 | 0.0905 | 0.8377 |
| rs4920359 | G | A | G | A | 1 | -0.0214 | 0.0025 | 3.10E-17 | -0.1217 | 0.0636 | 0.0557596 |
| rs55791371 | C | A | C | A | 19 | 0.0277 | 0.0038 | 1.80E-13 | -0.099 | 0.1013 | 0.3284 |
| rs56219219 | T | C | T | C | 4 | 0.0221 | 0.0037 | 1.65E-09 | -0.091 | 0.1093 | 0.4048 |
| rs56675301 | C | T | C | T | 1 | 0.0181 | 0.0028 | 4.50E-11 | -0.0379 | 0.0681 | 0.5777 |
| rs58542926 | T | C | T | C | 19 | 0.0448 | 0.0046 | 1.94E-22 | 0.0278 | 0.1234 | 0.8217 |
| rs62302166 | G | T | G | T | 4 | -0.162 | 0.0051 | 1.00E-200 | 0.0139 | 0.1348 | 0.9179 |
| rs6576006 | C | A | C | A | 14 | 0.0158 | 0.0026 | 1.06E-09 | -0.0313 | 0.0629 | 0.6191 |
| rs6600937 | A | G | A | G | 4 | 0.0208 | 0.0027 | 9.06E-15 | 0.0541 | 0.0624 | 0.386 |
| rs6754295 | G | T | G | T | 2 | 0.0162 | 0.0029 | 2.09E-08 | 0.0336 | 0.0683 | 0.6231 |
| rs6814839 | G | A | G | A | 4 | -0.1166 | 0.0048 | 7.73E-131 | 0.03 | 0.1122 | 0.7891 |
| rs71473824 | T | C | T | C | 11 | -0.0552 | 0.0068 | 3.68E-16 | -0.2885 | 0.1567 | 0.0655904 |
| rs7499892 | T | C | T | C | 16 | 0.0244 | 0.0031 | 8.50E-15 | 0.0487 | 0.0806 | 0.5456 |
| rs7652808 | G | T | G | T | 3 | -0.0171 | 0.0026 | 2.34E-11 | 0.0645 | 0.0686 | 0.3476 |
| rs79444700 | C | T | C | T | 4 | 0.0851 | 0.006 | 6.70E-46 | -0.0316 | 0.1708 | 0.8533 |
| rs8123293 | G | A | G | A | 20 | 0.0275 | 0.0038 | 4.41E-13 | 0.0859 | 0.0793 | 0.2791 |
| rs883541 | A | G | A | G | 17 | 0.0173 | 0.0029 | 2.61E-09 | -0.1027 | 0.0718 | 0.1527 |

| Supplementary table 7. Harmonized dataset of univariate Mendelian randomization for the effect of 25-hydroxyvitamin D on SLE with model 1 | | | | | | | | | | | |
| --- | --- | --- | --- | --- | --- | --- | --- | --- | --- | --- | --- |
|  |  |  |  |  |  | Exposure | | | Outcome | | |
| SNP | effect_allele.exposure | other_allele.exposure | effect_allele.outcome | other_allele.outcome | Chromosome | Effect | SE | P-value | Effect | SE | P-value |
| rs10017024 | C | T | C | T | 4 | -0.010936 | 0.001942 | 1.00E-200 | 0.106 | 0.0612 | 0.0832703 |
| rs10045097 | A | G | A | G | 5 | -0.010873 | 0.002001 | 1.00E-200 | 0.0811 | 0.0629 | 0.1974 |
| rs10070734 | C | T | C | T | 5 | 0.012 | 0.002136 | 1.00E-200 | 0.0425 | 0.0688 | 0.5374 |
| rs1022669 | G | A | G | A | 14 | -0.010409 | 0.001977 | 1.00E-200 | -0.0111 | 0.062 | 0.8584 |
| rs1026337 | T | A | T | A | 4 | -0.022376 | 0.003494 | 1.00E-200 | 0.0105 | 0.1071 | 0.9216 |
| rs1047891 | A | C | A | C | 2 | -0.014168 | 0.002088 | 1.00E-200 | 0.0245 | 0.0658 | 0.709999 |
| rs10518055 | G | T | G | T | 4 | -0.013606 | 0.002674 | 1.00E-200 | 0.1084 | 0.0782 | 0.1657 |
| rs10771085 | T | C | T | C | 12 | -0.010766 | 0.002012 | 1.00E-200 | -0.0035 | 0.063 | 0.9562 |
| rs10876448 | G | A | G | A | 12 | -0.013039 | 0.00257 | 1.00E-200 | -0.0075 | 0.0825 | 0.9273 |
| rs10888010 | C | T | C | T | 1 | -0.012255 | 0.001958 | 1.00E-200 | -0.0645 | 0.0615 | 0.2945 |
| rs11060406 | T | C | T | C | 12 | -0.027258 | 0.005251 | 1.00E-200 | 0.2606 | 0.1446 | 0.0714694 |
| rs11150592 | A | G | A | G | 16 | -0.014358 | 0.002738 | 1.00E-200 | -0.1169 | 0.0827 | 0.1573 |
| rs111757536 | A | G | A | G | 11 | 0.034417 | 0.006096 | 1.00E-200 | 0.0492 | 0.1333 | 0.712 |
| rs11209943 | G | A | G | A | 1 | -0.01042 | 0.001976 | 1.00E-200 | -0.005 | 0.0638 | 0.9374 |
| rs11227307 | A | G | A | G | 11 | 0.0115 | 0.002028 | 1.00E-200 | 0.0242 | 0.065 | 0.7094 |
| rs11253202 | C | T | C | T | 10 | 0.012053 | 0.002379 | 1.00E-200 | 0.0093 | 0.0911 | 0.9184 |
| rs114165281 | T | C | T | C | 4 | 0.037145 | 0.006062 | 1.00E-200 | -0.4248 | 0.2299 | 0.0646606 |
| rs114165349 | C | G | C | G | 1 | -0.035506 | 0.006506 | 1.00E-200 | -0.224 | 0.1654 | 0.1757 |
| rs114188086 | A | G | A | G | 4 | 0.027993 | 0.005322 | 1.00E-200 | -0.2004 | 0.1777 | 0.2593 |
| rs114952461 | C | T | C | T | 4 | -0.080618 | 0.00874 | 1.00E-200 | 0.0304 | 0.2209 | 0.8906 |
| rs1149555 | A | G | A | G | 7 | -0.012433 | 0.002464 | 1.00E-200 | -0.145 | 0.0853 | 0.0890594 |
| rs11582620 | G | A | G | A | 1 | -0.015454 | 0.003006 | 1.00E-200 | -0.0476 | 0.0945 | 0.614301 |
| rs11591147 | T | G | T | G | 1 | 0.046744 | 0.007316 | 1.00E-200 | 0.01 | 0.1682 | 0.9524 |
| rs11600578 | T | C | T | C | 11 | -0.047941 | 0.004953 | 1.00E-200 | -0.0526 | 0.1812 | 0.7715 |
| rs11602347 | G | C | G | C | 11 | 0.01011 | 0.001996 | 1.00E-200 | -0.0664 | 0.0645 | 0.3038 |
| rs11635491 | A | G | A | G | 15 | -0.021445 | 0.002215 | 1.00E-200 | -0.0285 | 0.069 | 0.6791 |
| rs11714324 | T | C | T | C | 3 | 0.013619 | 0.002615 | 1.00E-200 | 0.1113 | 0.09 | 0.2162 |
| rs117562170 | C | A | C | A | 12 | -0.032322 | 0.00477 | 1.00E-200 | -0.0409 | 0.0938 | 0.6629 |
| rs117939970 | C | T | C | T | 11 | -0.027276 | 0.005406 | 1.00E-200 | -0.0016 | 0.1693 | 0.9927 |
| rs118032309 | C | T | C | T | 11 | -0.044566 | 0.00873 | 1.00E-200 | 0.0502 | 0.162 | 0.7565 |
| rs11830764 | C | G | C | G | 12 | 0.021939 | 0.003907 | 1.00E-200 | 0.0269 | 0.1498 | 0.8576 |
| rs11885466 | T | C | T | C | 2 | -0.018832 | 0.003698 | 1.00E-200 | 0.1063 | 0.1044 | 0.3088 |
| rs11928368 | T | G | T | G | 3 | -0.012822 | 0.002083 | 1.00E-200 | 0.164 | 0.0627 | 0.0088889 |
| rs12035012 | A | C | A | C | 1 | 0.013916 | 0.002336 | 1.00E-200 | 0.0671 | 0.074 | 0.3644 |
| rs12423650 | A | G | A | G | 12 | -0.016074 | 0.003168 | 1.00E-200 | 0.0533 | 0.0923 | 0.5635 |
| rs12503220 | A | G | A | G | 4 | -0.013352 | 0.002479 | 1.00E-200 | -0.0264 | 0.0867 | 0.7604 |
| rs12546526 | C | T | C | T | 8 | 0.014686 | 0.002851 | 1.00E-200 | 0.0685 | 0.1113 | 0.5382 |
| rs12554549 | T | C | T | C | 9 | 0.020931 | 0.004003 | 1.00E-200 | -0.1434 | 0.1143 | 0.2097 |
| rs12797447 | T | A | T | A | 11 | 0.011627 | 0.002248 | 1.00E-200 | -0.0406 | 0.0662 | 0.5391 |
| rs12798050 | T | C | T | C | 11 | 0.107236 | 0.0026 | 1.00E-200 | 0.0872 | 0.0653 | 0.1818 |
| rs12800076 | T | G | T | G | 11 | -0.03196 | 0.006226 | 1.00E-200 | -0.3381 | 0.4751 | 0.4767 |
| rs12913937 | A | G | A | G | 15 | 0.010424 | 0.00205 | 1.00E-200 | -0.0494 | 0.0637 | 0.438 |
| rs12997900 | G | A | G | A | 2 | -0.011611 | 0.002221 | 1.00E-200 | 0.0365 | 0.0688 | 0.5953 |
| rs13065677 | T | C | T | C | 3 | 0.022753 | 0.004418 | 1.00E-200 | -0.1699 | 0.1607 | 0.2905 |
| rs13187496 | G | T | G | T | 5 | 0.01108 | 0.002055 | 1.00E-200 | 0.0318 | 0.064 | 0.6192 |
| rs13197862 | A | G | A | G | 6 | 0.014795 | 0.002899 | 1.00E-200 | -0.1312 | 0.1153 | 0.2552 |
| rs133075 | T | G | T | G | 22 | 0.010248 | 0.001971 | 1.00E-200 | -0.0583 | 0.0632 | 0.3558 |
| rs139047585 | T | C | T | C | 4 | -0.036021 | 0.007119 | 1.00E-200 | -0.1279 | 0.5618 | 0.8199 |
| rs141970801 | A | G | A | G | 2 | 0.027851 | 0.005003 | 1.00E-200 | -0.0616 | 0.1234 | 0.617701 |
| rs143375244 | G | A | G | A | 4 | -0.048176 | 0.008935 | 1.00E-200 | 0.2231 | 0.3458 | 0.5188 |
| rs143610797 | A | G | A | G | 20 | -0.036184 | 0.006996 | 1.00E-200 | -0.2348 | 0.2367 | 0.3211 |
| rs144366778 | T | C | T | C | 14 | 0.031113 | 0.005669 | 1.00E-200 | -0.3616 | 0.1737 | 0.0373499 |
| rs144628971 | A | G | A | G | 20 | 0.033496 | 0.005819 | 1.00E-200 | -0.3533 | 0.3346 | 0.291 |
| rs145662623 | A | G | A | G | 4 | 0.020312 | 0.004026 | 1.00E-200 | 0.5754 | 0.2263 | 0.0109901 |
| rs146735006 | T | C | T | C | 11 | -0.039794 | 0.007313 | 1.00E-200 | -0.2275 | 0.4661 | 0.6255 |
| rs148747986 | A | G | A | G | 4 | 0.04229 | 0.00729 | 1.00E-200 | 0.0878 | 0.2383 | 0.7124 |
| rs150057262 | G | C | G | C | 19 | 0.05113 | 0.009635 | 1.00E-200 | 0.211 | 0.1448 | 0.1449 |
| rs151302903 | C | G | C | G | 2 | -0.010745 | 0.002127 | 1.00E-200 | -0.0909 | 0.0647 | 0.1599 |
| rs1564366 | C | G | C | G | 12 | 0.011968 | 0.002378 | 1.00E-200 | -0.0545 | 0.0659 | 0.4081 |
| rs16830473 | C | T | C | T | 3 | 0.01756 | 0.003397 | 1.00E-200 | 0.0236 | 0.0954 | 0.8046 |
| rs1694929 | T | C | T | C | 3 | 0.009913 | 0.001969 | 1.00E-200 | -0.0307 | 0.0628 | 0.624901 |
| rs16961568 | G | A | G | A | 18 | -0.025821 | 0.004769 | 1.00E-200 | 0.1437 | 0.1516 | 0.3432 |
| rs16980051 | C | T | C | T | 19 | -0.009867 | 0.00195 | 1.00E-200 | 0.0257 | 0.0617 | 0.676901 |
| rs16998964 | T | C | T | C | 20 | 0.031026 | 0.004433 | 1.00E-200 | -0.0844 | 0.0929 | 0.3634 |
| rs17185287 | A | G | A | G | 15 | -0.01423 | 0.002674 | 1.00E-200 | -0.0646 | 0.073 | 0.3765 |
| rs17309874 | A | G | A | G | 11 | -0.011321 | 0.002204 | 1.00E-200 | -0.0155 | 0.0682 | 0.8198 |
| rs1800440 | C | T | C | T | 2 | -0.014006 | 0.002498 | 1.00E-200 | -0.079 | 0.0861 | 0.3588 |
| rs190343325 | C | T | C | T | 11 | -0.030062 | 0.005586 | 1.00E-200 | -0.0538 | 0.2061 | 0.793999 |
| rs1909585 | T | C | T | C | 3 | 0.0105 | 0.002071 | 1.00E-200 | -0.0215 | 0.0652 | 0.7417 |
| rs1943681 | T | A | T | A | 18 | -0.010345 | 0.002009 | 1.00E-200 | -0.1321 | 0.0642 | 0.0397603 |
| rs1957065 | G | C | G | C | 14 | 0.021307 | 0.003706 | 1.00E-200 | -0.1104 | 0.1426 | 0.4387 |
| rs2023910 | A | G | A | G | 7 | -0.01177 | 0.002221 | 1.00E-200 | 0.0063 | 0.0691 | 0.9277 |
| rs2043084 | C | G | C | G | 15 | -0.020735 | 0.002333 | 1.00E-200 | 0.0776 | 0.0743 | 0.296 |
| rs2068190 | A | G | A | G | 5 | 0.010237 | 0.001961 | 1.00E-200 | -0.0213 | 0.0622 | 0.732099 |
| rs2071408 | A | G | A | G | 14 | 0.011625 | 0.002034 | 1.00E-200 | -0.0546 | 0.0634 | 0.3894 |
| rs2207132 | A | G | A | G | 20 | -0.029118 | 0.005507 | 1.00E-200 | 0.0709 | 0.1238 | 0.566601 |
| rs2229742 | C | G | C | G | 21 | -0.025738 | 0.00319 | 1.00E-200 | -0.1847 | 0.097 | 0.0568696 |
| rs2246281 | T | G | T | G | 6 | -0.012434 | 0.002328 | 1.00E-200 | -0.0434 | 0.0664 | 0.513499 |
| rs2282619 | C | T | C | T | 11 | 0.084799 | 0.004822 | 1.00E-200 | 0.2174 | 0.1277 | 0.0887892 |
| rs2295659 | T | G | T | G | 14 | -0.014828 | 0.002923 | 1.00E-200 | -0.0981 | 0.0829 | 0.2369 |
| rs2304634 | T | C | T | C | 16 | 0.010611 | 0.002106 | 1.00E-200 | 0.0735 | 0.0678 | 0.278 |
| rs2306390 | T | C | T | C | 12 | -0.01157 | 0.002223 | 1.00E-200 | -0.0066 | 0.0671 | 0.9215 |
| rs2519093 | T | C | T | C | 9 | -0.014952 | 0.002518 | 1.00E-200 | 0.0172 | 0.0762 | 0.8214 |
| rs2607838 | G | A | G | A | 10 | -0.020233 | 0.003934 | 1.00E-200 | 0.1029 | 0.1659 | 0.5349 |
| rs2642438 | G | A | G | A | 1 | -0.013439 | 0.002122 | 1.00E-200 | -0.0179 | 0.068 | 0.791799 |
| rs2843128 | G | A | G | A | 1 | 0.009897 | 0.001938 | 1.00E-200 | 0.0268 | 0.0617 | 0.6648 |
| rs2999559 | C | A | C | A | 1 | -0.011466 | 0.002234 | 1.00E-200 | -0.0446 | 0.0644 | 0.4888 |
| rs336605 | T | G | T | G | 3 | -0.0111 | 0.002191 | 1.00E-200 | -2.00E-04 | 0.0656 | 0.998 |
| rs34560261 | T | C | T | C | 15 | 0.013552 | 0.002657 | 1.00E-200 | -0.0322 | 0.0886 | 0.716401 |
| rs34871842 | A | G | A | G | 19 | 0.018981 | 0.003494 | 1.00E-200 | -0.1296 | 0.1132 | 0.2524 |
| rs3745535 | C | A | C | A | 19 | 0.010447 | 0.002035 | 1.00E-200 | 0.0163 | 0.0633 | 0.7966 |
| rs3761077 | T | G | T | G | 19 | 0.017336 | 0.0031 | 1.00E-200 | -0.08 | 0.1104 | 0.4688 |
| rs3814995 | T | C | T | C | 19 | -0.014733 | 0.002109 | 1.00E-200 | -0.0698 | 0.0648 | 0.2816 |
| rs3817588 | C | T | C | T | 2 | 0.014713 | 0.002469 | 1.00E-200 | 0.0354 | 0.071 | 0.6183 |
| rs41266415 | T | A | T | A | 1 | 0.013119 | 0.002367 | 1.00E-200 | -0.0467 | 0.081 | 0.5642 |
| rs41290120 | A | G | A | G | 19 | 0.028026 | 0.004488 | 1.00E-200 | -0.0298 | 0.1776 | 0.8669 |
| rs4364259 | A | G | A | G | 4 | 0.015798 | 0.002422 | 1.00E-200 | 0.0805 | 0.0742 | 0.2783 |
| rs4466239 | G | A | G | A | 6 | 0.01098 | 0.002023 | 1.00E-200 | 0.0769 | 0.0637 | 0.2275 |
| rs4553272 | T | C | T | C | 10 | -0.01036 | 0.001958 | 1.00E-200 | 0.0032 | 0.0617 | 0.9585 |
| rs4631704 | T | C | T | C | 1 | 0.010978 | 0.002012 | 1.00E-200 | -0.007 | 0.0617 | 0.9101 |
| rs4663871 | A | G | A | G | 2 | -0.018796 | 0.002313 | 1.00E-200 | -0.0558 | 0.075 | 0.4564 |
| rs484195 | G | A | G | A | 19 | -0.014991 | 0.002045 | 1.00E-200 | -0.0695 | 0.0662 | 0.2941 |
| rs4971020 | C | T | C | T | 1 | 0.010532 | 0.002036 | 1.00E-200 | -0.1104 | 0.0637 | 0.08314 |
| rs512083 | C | T | C | T | 1 | 0.009979 | 0.001948 | 1.00E-200 | -0.0841 | 0.0613 | 0.1701 |
| rs513533 | G | A | G | A | 11 | -0.020039 | 0.003224 | 1.00E-200 | -0.0068 | 0.0798 | 0.9316 |
| rs537089 | G | C | G | C | 11 | -0.021591 | 0.003581 | 1.00E-200 | 0.0772 | 0.108 | 0.4749 |
| rs56059718 | A | C | A | C | 15 | -0.012535 | 0.002474 | 1.00E-200 | -0.0273 | 0.077 | 0.7227 |
| rs56158152 | T | G | T | G | 16 | 0.01084 | 0.002114 | 1.00E-200 | -0.0939 | 0.0633 | 0.1383 |
| rs6018088 | A | C | A | C | 20 | -0.015435 | 0.003044 | 1.00E-200 | -0.1479 | 0.0873 | 0.0903504 |
| rs60500353 | T | C | T | C | 1 | 0.01532 | 0.00273 | 1.00E-200 | -0.133 | 0.102 | 0.1921 |
| rs60954647 | C | T | C | T | 11 | -0.01004 | 0.001947 | 1.00E-200 | -0.0385 | 0.0619 | 0.5337 |
| rs62215568 | A | G | A | G | 20 | 0.035014 | 0.006609 | 1.00E-200 | 0.0622 | 0.1504 | 0.679401 |
| rs62299542 | A | G | A | G | 4 | 0.023662 | 0.002932 | 1.00E-200 | 0.0266 | 0.1078 | 0.8054 |
| rs62493791 | G | T | G | T | 8 | 0.01207 | 0.002297 | 1.00E-200 | 0.067 | 0.0766 | 0.3814 |
| rs62568181 | C | T | C | T | 9 | 0.016228 | 0.003192 | 1.00E-200 | -0.0861 | 0.112 | 0.4424 |
| rs6657811 | T | A | T | A | 1 | 0.015448 | 0.002878 | 1.00E-200 | 0.0369 | 0.101 | 0.714801 |
| rs673335 | C | T | C | T | 11 | 0.016497 | 0.00264 | 1.00E-200 | 0.0189 | 0.0713 | 0.7905 |
| rs6750649 | G | T | G | T | 2 | -0.010261 | 0.002006 | 1.00E-200 | 0.0346 | 0.0626 | 0.580301 |
| rs68033110 | A | G | A | G | 18 | -0.011617 | 0.002294 | 1.00E-200 | -0.0845 | 0.0785 | 0.282 |
| rs6970645 | G | C | G | C | 7 | 0.011468 | 0.002265 | 1.00E-200 | -0.1715 | 0.0733 | 0.01923 |
| rs6985620 | C | T | C | T | 8 | 0.010643 | 0.002061 | 1.00E-200 | -0.0531 | 0.063 | 0.3998 |
| rs7027254 | C | T | C | T | 9 | 0.014431 | 0.002774 | 1.00E-200 | -0.0427 | 0.0812 | 0.5994 |
| rs7189954 | T | C | T | C | 16 | -0.010407 | 0.002034 | 1.00E-200 | 0.1581 | 0.0644 | 0.0141198 |
| rs7258060 | T | A | T | A | 19 | -0.010693 | 0.002082 | 1.00E-200 | -0.0489 | 0.0636 | 0.4415 |
| rs72651860 | A | G | A | G | 4 | -0.113727 | 0.010222 | 1.00E-200 | -0.7027 | 0.2904 | 0.01554 |
| rs72680100 | G | A | G | A | 14 | -0.026781 | 0.005057 | 1.00E-200 | -0.3243 | 0.2057 | 0.1149 |
| rs72739147 | T | A | T | A | 15 | 0.015955 | 0.002903 | 1.00E-200 | 0.0411 | 0.103 | 0.69 |
| rs7297538 | C | T | C | T | 12 | 0.010802 | 0.002032 | 1.00E-200 | 0.0186 | 0.0631 | 0.767701 |
| rs7330 | A | C | A | C | 12 | -0.011137 | 0.002013 | 1.00E-200 | -0.0091 | 0.0648 | 0.8884 |
| rs7419651 | A | G | A | G | 2 | -0.01788 | 0.003005 | 1.00E-200 | -0.0324 | 0.0865 | 0.7077 |
| rs74342059 | T | C | T | C | 11 | 0.030593 | 0.005458 | 1.00E-200 | 0.3575 | 0.1907 | 0.0609102 |
| rs7511513 | T | C | T | C | 22 | -0.010402 | 0.002064 | 1.00E-200 | 0.0595 | 0.0618 | 0.3358 |
| rs75865451 | A | G | A | G | 6 | -0.016308 | 0.003176 | 1.00E-200 | 0.0788 | 0.108 | 0.4657 |
| rs76183418 | C | T | C | T | 3 | 0.013077 | 0.002593 | 1.00E-200 | -0.0133 | 0.073 | 0.8555 |
| rs7640441 | A | C | A | C | 3 | 0.013242 | 0.002293 | 1.00E-200 | 0.0703 | 0.0674 | 0.2971 |
| rs7651161 | C | T | C | T | 3 | -0.011027 | 0.001981 | 1.00E-200 | 0.1411 | 0.0617 | 0.02223 |
| rs76935526 | T | C | T | C | 2 | -0.013302 | 0.00254 | 1.00E-200 | -0.0252 | 0.0819 | 0.758099 |
| rs77194050 | G | A | G | A | 16 | 0.023163 | 0.004447 | 1.00E-200 | 0.203 | 0.1558 | 0.1926 |
| rs77257135 | A | C | A | C | 4 | 0.047954 | 0.007699 | 1.00E-200 | -0.1272 | 0.2001 | 0.5248 |
| rs77885114 | A | G | A | G | 1 | -0.016609 | 0.00329 | 1.00E-200 | 0.0184 | 0.0984 | 0.852 |
| rs7794142 | A | G | A | G | 7 | 0.011397 | 0.001944 | 1.00E-200 | 0.0146 | 0.0612 | 0.8109 |
| rs78117488 | T | C | T | C | 12 | 0.021296 | 0.004118 | 1.00E-200 | -0.0082 | 0.109 | 0.9404 |
| rs7900214 | A | G | A | G | 10 | 0.011705 | 0.002182 | 1.00E-200 | 0.0544 | 0.0705 | 0.4408 |
| rs7924820 | A | G | A | G | 11 | -0.023968 | 0.00471 | 1.00E-200 | -0.0553 | 0.126 | 0.660601 |
| rs79687284 | C | G | C | G | 1 | -0.02728 | 0.005411 | 1.00E-200 | -0.0901 | 0.2274 | 0.6921 |
| rs7981402 | A | G | A | G | 13 | 0.010448 | 0.002055 | 1.00E-200 | 0.0065 | 0.066 | 0.922 |
| rs800531 | C | T | C | T | 8 | -0.012752 | 0.002345 | 1.00E-200 | -0.0371 | 0.0727 | 0.6101 |
| rs80067728 | G | A | G | A | 4 | 0.05381 | 0.008794 | 1.00E-200 | -0.2298 | 0.2004 | 0.2515 |
| rs80204526 | A | C | A | C | 18 | -0.048218 | 0.00942 | 1.00E-200 | 0.2393 | 0.4274 | 0.5756 |
| rs897438 | G | A | G | A | 18 | -0.011957 | 0.002328 | 1.00E-200 | -0.0163 | 0.0742 | 0.8256 |
| rs942380 | G | A | G | A | 6 | 0.011434 | 0.001981 | 1.00E-200 | -0.0611 | 0.0675 | 0.3655 |
| rs951914 | C | G | C | G | 8 | -0.011397 | 0.002161 | 1.00E-200 | -0.01 | 0.0644 | 0.877 |
| rs9536961 | G | A | G | A | 13 | -0.011759 | 0.00207 | 1.00E-200 | -0.0466 | 0.0637 | 0.4648 |
| rs982890 | C | T | C | T | 11 | 0.013959 | 0.001939 | 1.00E-200 | 0.0524 | 0.0613 | 0.3928 |
| rs9861009 | C | T | C | T | 3 | 0.012425 | 0.002185 | 1.00E-200 | 0.0725 | 0.0698 | 0.2992 |
| rs9889884 | C | T | C | T | 17 | 0.013186 | 0.002291 | 1.00E-200 | -0.1324 | 0.0799 | 0.0976697 |
| rs9926530 | G | T | G | T | 16 | 0.022162 | 0.004329 | 1.00E-200 | -0.0045 | 0.1258 | 0.9717 |
| rs9928757 | C | G | C | G | 16 | -0.013101 | 0.002423 | 1.00E-200 | 0.0723 | 0.0701 | 0.3025 |
| rs9989419 | G | A | G | A | 16 | -0.012128 | 0.001995 | 1.00E-200 | 0.0298 | 0.0628 | 0.6356 |
| rs9998874 | G | T | G | T | 4 | 0.026735 | 0.00395 | 1.00E-200 | -0.0156 | 0.139 | 0.9106 |

| Supplementary table 8. Harmonized dataset of univariate Mendelian randomization for the effect of 25-hydroxyvitamin D on SLE with model 2 | | | | | | | | | | | |
| --- | --- | --- | --- | --- | --- | --- | --- | --- | --- | --- | --- |
|  |  |  |  |  |  | Exposure | | | Outcome | | |
| SNP | effect_allele.exposure | other_allele.exposure | effect_allele.outcome | other_allele.outcome | Chromosome | Effect | SE | P-value | Effect | SE | P-value |
| rs10017024 | C | T | C | T | 4 | -0.010936 | 0.001942 | 1.00E-200 | 0.106 | 0.0612 | 0.0832703 |
| rs10045097 | A | G | A | G | 5 | -0.010873 | 0.002001 | 1.00E-200 | 0.0811 | 0.0629 | 0.1974 |
| rs10070734 | C | T | C | T | 5 | 0.012 | 0.002136 | 1.00E-200 | 0.0425 | 0.0688 | 0.5374 |
| rs1022669 | G | A | G | A | 14 | -0.010409 | 0.001977 | 1.00E-200 | -0.0111 | 0.062 | 0.8584 |
| rs1026337 | T | A | T | A | 4 | -0.022376 | 0.003494 | 1.00E-200 | 0.0105 | 0.1071 | 0.9216 |
| rs10518055 | G | T | G | T | 4 | -0.013606 | 0.002674 | 1.00E-200 | 0.1084 | 0.0782 | 0.1657 |
| rs10771085 | T | C | T | C | 12 | -0.010766 | 0.002012 | 1.00E-200 | -0.0035 | 0.063 | 0.9562 |
| rs10876448 | G | A | G | A | 12 | -0.013039 | 0.00257 | 1.00E-200 | -0.0075 | 0.0825 | 0.9273 |
| rs10888010 | C | T | C | T | 1 | -0.012255 | 0.001958 | 1.00E-200 | -0.0645 | 0.0615 | 0.2945 |
| rs11060406 | T | C | T | C | 12 | -0.027258 | 0.005251 | 1.00E-200 | 0.2606 | 0.1446 | 0.0714694 |
| rs11150592 | A | G | A | G | 16 | -0.014358 | 0.002738 | 1.00E-200 | -0.1169 | 0.0827 | 0.1573 |
| rs111757536 | A | G | A | G | 11 | 0.034417 | 0.006096 | 1.00E-200 | 0.0492 | 0.1333 | 0.712 |
| rs11209943 | G | A | G | A | 1 | -0.01042 | 0.001976 | 1.00E-200 | -0.005 | 0.0638 | 0.9374 |
| rs11227307 | A | G | A | G | 11 | 0.0115 | 0.002028 | 1.00E-200 | 0.0242 | 0.065 | 0.7094 |
| rs11253202 | C | T | C | T | 10 | 0.012053 | 0.002379 | 1.00E-200 | 0.0093 | 0.0911 | 0.9184 |
| rs114165281 | T | C | T | C | 4 | 0.037145 | 0.006062 | 1.00E-200 | -0.4248 | 0.2299 | 0.0646606 |
| rs114165349 | C | G | C | G | 1 | -0.035506 | 0.006506 | 1.00E-200 | -0.224 | 0.1654 | 0.1757 |
| rs114188086 | A | G | A | G | 4 | 0.027993 | 0.005322 | 1.00E-200 | -0.2004 | 0.1777 | 0.2593 |
| rs114952461 | C | T | C | T | 4 | -0.080618 | 0.00874 | 1.00E-200 | 0.0304 | 0.2209 | 0.8906 |
| rs1149555 | A | G | A | G | 7 | -0.012433 | 0.002464 | 1.00E-200 | -0.145 | 0.0853 | 0.0890594 |
| rs11582620 | G | A | G | A | 1 | -0.015454 | 0.003006 | 1.00E-200 | -0.0476 | 0.0945 | 0.614301 |
| rs11591147 | T | G | T | G | 1 | 0.046744 | 0.007316 | 1.00E-200 | 0.01 | 0.1682 | 0.9524 |
| rs11600578 | T | C | T | C | 11 | -0.047941 | 0.004953 | 1.00E-200 | -0.0526 | 0.1812 | 0.7715 |
| rs11602347 | G | C | G | C | 11 | 0.01011 | 0.001996 | 1.00E-200 | -0.0664 | 0.0645 | 0.3038 |
| rs11635491 | A | G | A | G | 15 | -0.021445 | 0.002215 | 1.00E-200 | -0.0285 | 0.069 | 0.6791 |
| rs11714324 | T | C | T | C | 3 | 0.013619 | 0.002615 | 1.00E-200 | 0.1113 | 0.09 | 0.2162 |
| rs117562170 | C | A | C | A | 12 | -0.032322 | 0.00477 | 1.00E-200 | -0.0409 | 0.0938 | 0.6629 |
| rs117939970 | C | T | C | T | 11 | -0.027276 | 0.005406 | 1.00E-200 | -0.0016 | 0.1693 | 0.9927 |
| rs118032309 | C | T | C | T | 11 | -0.044566 | 0.00873 | 1.00E-200 | 0.0502 | 0.162 | 0.7565 |
| rs11830764 | C | G | C | G | 12 | 0.021939 | 0.003907 | 1.00E-200 | 0.0269 | 0.1498 | 0.8576 |
| rs11885466 | T | C | T | C | 2 | -0.018832 | 0.003698 | 1.00E-200 | 0.1063 | 0.1044 | 0.3088 |
| rs11928368 | T | G | T | G | 3 | -0.012822 | 0.002083 | 1.00E-200 | 0.164 | 0.0627 | 0.0088889 |
| rs12035012 | A | C | A | C | 1 | 0.013916 | 0.002336 | 1.00E-200 | 0.0671 | 0.074 | 0.3644 |
| rs12423650 | A | G | A | G | 12 | -0.016074 | 0.003168 | 1.00E-200 | 0.0533 | 0.0923 | 0.5635 |
| rs12546526 | C | T | C | T | 8 | 0.014686 | 0.002851 | 1.00E-200 | 0.0685 | 0.1113 | 0.5382 |
| rs12554549 | T | C | T | C | 9 | 0.020931 | 0.004003 | 1.00E-200 | -0.1434 | 0.1143 | 0.2097 |
| rs12797447 | T | A | T | A | 11 | 0.011627 | 0.002248 | 1.00E-200 | -0.0406 | 0.0662 | 0.5391 |
| rs12798050 | T | C | T | C | 11 | 0.107236 | 0.0026 | 1.00E-200 | 0.0872 | 0.0653 | 0.1818 |
| rs12800076 | T | G | T | G | 11 | -0.03196 | 0.006226 | 1.00E-200 | -0.3381 | 0.4751 | 0.4767 |
| rs12913937 | A | G | A | G | 15 | 0.010424 | 0.00205 | 1.00E-200 | -0.0494 | 0.0637 | 0.438 |
| rs13065677 | T | C | T | C | 3 | 0.022753 | 0.004418 | 1.00E-200 | -0.1699 | 0.1607 | 0.2905 |
| rs13187496 | G | T | G | T | 5 | 0.01108 | 0.002055 | 1.00E-200 | 0.0318 | 0.064 | 0.6192 |
| rs13197862 | A | G | A | G | 6 | 0.014795 | 0.002899 | 1.00E-200 | -0.1312 | 0.1153 | 0.2552 |
| rs133075 | T | G | T | G | 22 | 0.010248 | 0.001971 | 1.00E-200 | -0.0583 | 0.0632 | 0.3558 |
| rs139047585 | T | C | T | C | 4 | -0.036021 | 0.007119 | 1.00E-200 | -0.1279 | 0.5618 | 0.8199 |
| rs141970801 | A | G | A | G | 2 | 0.027851 | 0.005003 | 1.00E-200 | -0.0616 | 0.1234 | 0.617701 |
| rs143375244 | G | A | G | A | 4 | -0.048176 | 0.008935 | 1.00E-200 | 0.2231 | 0.3458 | 0.5188 |
| rs143610797 | A | G | A | G | 20 | -0.036184 | 0.006996 | 1.00E-200 | -0.2348 | 0.2367 | 0.3211 |
| rs144366778 | T | C | T | C | 14 | 0.031113 | 0.005669 | 1.00E-200 | -0.3616 | 0.1737 | 0.0373499 |
| rs144628971 | A | G | A | G | 20 | 0.033496 | 0.005819 | 1.00E-200 | -0.3533 | 0.3346 | 0.291 |
| rs145662623 | A | G | A | G | 4 | 0.020312 | 0.004026 | 1.00E-200 | 0.5754 | 0.2263 | 0.0109901 |
| rs146735006 | T | C | T | C | 11 | -0.039794 | 0.007313 | 1.00E-200 | -0.2275 | 0.4661 | 0.6255 |
| rs148747986 | A | G | A | G | 4 | 0.04229 | 0.00729 | 1.00E-200 | 0.0878 | 0.2383 | 0.7124 |
| rs150057262 | G | C | G | C | 19 | 0.05113 | 0.009635 | 1.00E-200 | 0.211 | 0.1448 | 0.1449 |
| rs151302903 | C | G | C | G | 2 | -0.010745 | 0.002127 | 1.00E-200 | -0.0909 | 0.0647 | 0.1599 |
| rs1564366 | C | G | C | G | 12 | 0.011968 | 0.002378 | 1.00E-200 | -0.0545 | 0.0659 | 0.4081 |
| rs16830473 | C | T | C | T | 3 | 0.01756 | 0.003397 | 1.00E-200 | 0.0236 | 0.0954 | 0.8046 |
| rs1694929 | T | C | T | C | 3 | 0.009913 | 0.001969 | 1.00E-200 | -0.0307 | 0.0628 | 0.624901 |
| rs16961568 | G | A | G | A | 18 | -0.025821 | 0.004769 | 1.00E-200 | 0.1437 | 0.1516 | 0.3432 |
| rs16998964 | T | C | T | C | 20 | 0.031026 | 0.004433 | 1.00E-200 | -0.0844 | 0.0929 | 0.3634 |
| rs17185287 | A | G | A | G | 15 | -0.01423 | 0.002674 | 1.00E-200 | -0.0646 | 0.073 | 0.3765 |
| rs1800440 | C | T | C | T | 2 | -0.014006 | 0.002498 | 1.00E-200 | -0.079 | 0.0861 | 0.3588 |
| rs190343325 | C | T | C | T | 11 | -0.030062 | 0.005586 | 1.00E-200 | -0.0538 | 0.2061 | 0.793999 |
| rs1909585 | T | C | T | C | 3 | 0.0105 | 0.002071 | 1.00E-200 | -0.0215 | 0.0652 | 0.7417 |
| rs1943681 | T | A | T | A | 18 | -0.010345 | 0.002009 | 1.00E-200 | -0.1321 | 0.0642 | 0.0397603 |
| rs1957065 | G | C | G | C | 14 | 0.021307 | 0.003706 | 1.00E-200 | -0.1104 | 0.1426 | 0.4387 |
| rs2023910 | A | G | A | G | 7 | -0.01177 | 0.002221 | 1.00E-200 | 0.0063 | 0.0691 | 0.9277 |
| rs2043084 | C | G | C | G | 15 | -0.020735 | 0.002333 | 1.00E-200 | 0.0776 | 0.0743 | 0.296 |
| rs2068190 | A | G | A | G | 5 | 0.010237 | 0.001961 | 1.00E-200 | -0.0213 | 0.0622 | 0.732099 |
| rs2071408 | A | G | A | G | 14 | 0.011625 | 0.002034 | 1.00E-200 | -0.0546 | 0.0634 | 0.3894 |
| rs2207132 | A | G | A | G | 20 | -0.029118 | 0.005507 | 1.00E-200 | 0.0709 | 0.1238 | 0.566601 |
| rs2229742 | C | G | C | G | 21 | -0.025738 | 0.00319 | 1.00E-200 | -0.1847 | 0.097 | 0.0568696 |
| rs2246281 | T | G | T | G | 6 | -0.012434 | 0.002328 | 1.00E-200 | -0.0434 | 0.0664 | 0.513499 |
| rs2282619 | C | T | C | T | 11 | 0.084799 | 0.004822 | 1.00E-200 | 0.2174 | 0.1277 | 0.0887892 |
| rs2304634 | T | C | T | C | 16 | 0.010611 | 0.002106 | 1.00E-200 | 0.0735 | 0.0678 | 0.278 |
| rs2306390 | T | C | T | C | 12 | -0.01157 | 0.002223 | 1.00E-200 | -0.0066 | 0.0671 | 0.9215 |
| rs2607838 | G | A | G | A | 10 | -0.020233 | 0.003934 | 1.00E-200 | 0.1029 | 0.1659 | 0.5349 |
| rs2642438 | G | A | G | A | 1 | -0.013439 | 0.002122 | 1.00E-200 | -0.0179 | 0.068 | 0.791799 |
| rs2843128 | G | A | G | A | 1 | 0.009897 | 0.001938 | 1.00E-200 | 0.0268 | 0.0617 | 0.6648 |
| rs2999559 | C | A | C | A | 1 | -0.011466 | 0.002234 | 1.00E-200 | -0.0446 | 0.0644 | 0.4888 |
| rs34560261 | T | C | T | C | 15 | 0.013552 | 0.002657 | 1.00E-200 | -0.0322 | 0.0886 | 0.716401 |
| rs34871842 | A | G | A | G | 19 | 0.018981 | 0.003494 | 1.00E-200 | -0.1296 | 0.1132 | 0.2524 |
| rs3745535 | C | A | C | A | 19 | 0.010447 | 0.002035 | 1.00E-200 | 0.0163 | 0.0633 | 0.7966 |
| rs3761077 | T | G | T | G | 19 | 0.017336 | 0.0031 | 1.00E-200 | -0.08 | 0.1104 | 0.4688 |
| rs3814995 | T | C | T | C | 19 | -0.014733 | 0.002109 | 1.00E-200 | -0.0698 | 0.0648 | 0.2816 |
| rs3817588 | C | T | C | T | 2 | 0.014713 | 0.002469 | 1.00E-200 | 0.0354 | 0.071 | 0.6183 |
| rs41266415 | T | A | T | A | 1 | 0.013119 | 0.002367 | 1.00E-200 | -0.0467 | 0.081 | 0.5642 |
| rs41290120 | A | G | A | G | 19 | 0.028026 | 0.004488 | 1.00E-200 | -0.0298 | 0.1776 | 0.8669 |
| rs4364259 | A | G | A | G | 4 | 0.015798 | 0.002422 | 1.00E-200 | 0.0805 | 0.0742 | 0.2783 |
| rs4466239 | G | A | G | A | 6 | 0.01098 | 0.002023 | 1.00E-200 | 0.0769 | 0.0637 | 0.2275 |
| rs4631704 | T | C | T | C | 1 | 0.010978 | 0.002012 | 1.00E-200 | -0.007 | 0.0617 | 0.9101 |
| rs4663871 | A | G | A | G | 2 | -0.018796 | 0.002313 | 1.00E-200 | -0.0558 | 0.075 | 0.4564 |
| rs4971020 | C | T | C | T | 1 | 0.010532 | 0.002036 | 1.00E-200 | -0.1104 | 0.0637 | 0.08314 |
| rs513533 | G | A | G | A | 11 | -0.020039 | 0.003224 | 1.00E-200 | -0.0068 | 0.0798 | 0.9316 |
| rs537089 | G | C | G | C | 11 | -0.021591 | 0.003581 | 1.00E-200 | 0.0772 | 0.108 | 0.4749 |
| rs56059718 | A | C | A | C | 15 | -0.012535 | 0.002474 | 1.00E-200 | -0.0273 | 0.077 | 0.7227 |
| rs56158152 | T | G | T | G | 16 | 0.01084 | 0.002114 | 1.00E-200 | -0.0939 | 0.0633 | 0.1383 |
| rs6018088 | A | C | A | C | 20 | -0.015435 | 0.003044 | 1.00E-200 | -0.1479 | 0.0873 | 0.0903504 |
| rs60500353 | T | C | T | C | 1 | 0.01532 | 0.00273 | 1.00E-200 | -0.133 | 0.102 | 0.1921 |
| rs60954647 | C | T | C | T | 11 | -0.01004 | 0.001947 | 1.00E-200 | -0.0385 | 0.0619 | 0.5337 |
| rs62215568 | A | G | A | G | 20 | 0.035014 | 0.006609 | 1.00E-200 | 0.0622 | 0.1504 | 0.679401 |
| rs62299542 | A | G | A | G | 4 | 0.023662 | 0.002932 | 1.00E-200 | 0.0266 | 0.1078 | 0.8054 |
| rs62493791 | G | T | G | T | 8 | 0.01207 | 0.002297 | 1.00E-200 | 0.067 | 0.0766 | 0.3814 |
| rs62568181 | C | T | C | T | 9 | 0.016228 | 0.003192 | 1.00E-200 | -0.0861 | 0.112 | 0.4424 |
| rs6657811 | T | A | T | A | 1 | 0.015448 | 0.002878 | 1.00E-200 | 0.0369 | 0.101 | 0.714801 |
| rs673335 | C | T | C | T | 11 | 0.016497 | 0.00264 | 1.00E-200 | 0.0189 | 0.0713 | 0.7905 |
| rs6750649 | G | T | G | T | 2 | -0.010261 | 0.002006 | 1.00E-200 | 0.0346 | 0.0626 | 0.580301 |
| rs68033110 | A | G | A | G | 18 | -0.011617 | 0.002294 | 1.00E-200 | -0.0845 | 0.0785 | 0.282 |
| rs6985620 | C | T | C | T | 8 | 0.010643 | 0.002061 | 1.00E-200 | -0.0531 | 0.063 | 0.3998 |
| rs7027254 | C | T | C | T | 9 | 0.014431 | 0.002774 | 1.00E-200 | -0.0427 | 0.0812 | 0.5994 |
| rs7189954 | T | C | T | C | 16 | -0.010407 | 0.002034 | 1.00E-200 | 0.1581 | 0.0644 | 0.0141198 |
| rs7258060 | T | A | T | A | 19 | -0.010693 | 0.002082 | 1.00E-200 | -0.0489 | 0.0636 | 0.4415 |
| rs72651860 | A | G | A | G | 4 | -0.113727 | 0.010222 | 1.00E-200 | -0.7027 | 0.2904 | 0.01554 |
| rs72680100 | G | A | G | A | 14 | -0.026781 | 0.005057 | 1.00E-200 | -0.3243 | 0.2057 | 0.1149 |
| rs72739147 | T | A | T | A | 15 | 0.015955 | 0.002903 | 1.00E-200 | 0.0411 | 0.103 | 0.69 |
| rs7297538 | C | T | C | T | 12 | 0.010802 | 0.002032 | 1.00E-200 | 0.0186 | 0.0631 | 0.767701 |
| rs7330 | A | C | A | C | 12 | -0.011137 | 0.002013 | 1.00E-200 | -0.0091 | 0.0648 | 0.8884 |
| rs7419651 | A | G | A | G | 2 | -0.01788 | 0.003005 | 1.00E-200 | -0.0324 | 0.0865 | 0.7077 |
| rs74342059 | T | C | T | C | 11 | 0.030593 | 0.005458 | 1.00E-200 | 0.3575 | 0.1907 | 0.0609102 |
| rs7511513 | T | C | T | C | 22 | -0.010402 | 0.002064 | 1.00E-200 | 0.0595 | 0.0618 | 0.3358 |
| rs75865451 | A | G | A | G | 6 | -0.016308 | 0.003176 | 1.00E-200 | 0.0788 | 0.108 | 0.4657 |
| rs76183418 | C | T | C | T | 3 | 0.013077 | 0.002593 | 1.00E-200 | -0.0133 | 0.073 | 0.8555 |
| rs7640441 | A | C | A | C | 3 | 0.013242 | 0.002293 | 1.00E-200 | 0.0703 | 0.0674 | 0.2971 |
| rs7651161 | C | T | C | T | 3 | -0.011027 | 0.001981 | 1.00E-200 | 0.1411 | 0.0617 | 0.02223 |
| rs76935526 | T | C | T | C | 2 | -0.013302 | 0.00254 | 1.00E-200 | -0.0252 | 0.0819 | 0.758099 |
| rs77194050 | G | A | G | A | 16 | 0.023163 | 0.004447 | 1.00E-200 | 0.203 | 0.1558 | 0.1926 |
| rs77257135 | A | C | A | C | 4 | 0.047954 | 0.007699 | 1.00E-200 | -0.1272 | 0.2001 | 0.5248 |
| rs77885114 | A | G | A | G | 1 | -0.016609 | 0.00329 | 1.00E-200 | 0.0184 | 0.0984 | 0.852 |
| rs7794142 | A | G | A | G | 7 | 0.011397 | 0.001944 | 1.00E-200 | 0.0146 | 0.0612 | 0.8109 |
| rs78117488 | T | C | T | C | 12 | 0.021296 | 0.004118 | 1.00E-200 | -0.0082 | 0.109 | 0.9404 |
| rs7900214 | A | G | A | G | 10 | 0.011705 | 0.002182 | 1.00E-200 | 0.0544 | 0.0705 | 0.4408 |
| rs7924820 | A | G | A | G | 11 | -0.023968 | 0.00471 | 1.00E-200 | -0.0553 | 0.126 | 0.660601 |
| rs79687284 | C | G | C | G | 1 | -0.02728 | 0.005411 | 1.00E-200 | -0.0901 | 0.2274 | 0.6921 |
| rs7981402 | A | G | A | G | 13 | 0.010448 | 0.002055 | 1.00E-200 | 0.0065 | 0.066 | 0.922 |
| rs80067728 | G | A | G | A | 4 | 0.05381 | 0.008794 | 1.00E-200 | -0.2298 | 0.2004 | 0.2515 |
| rs80204526 | A | C | A | C | 18 | -0.048218 | 0.00942 | 1.00E-200 | 0.2393 | 0.4274 | 0.5756 |
| rs897438 | G | A | G | A | 18 | -0.011957 | 0.002328 | 1.00E-200 | -0.0163 | 0.0742 | 0.8256 |
| rs942380 | G | A | G | A | 6 | 0.011434 | 0.001981 | 1.00E-200 | -0.0611 | 0.0675 | 0.3655 |
| rs951914 | C | G | C | G | 8 | -0.011397 | 0.002161 | 1.00E-200 | -0.01 | 0.0644 | 0.877 |
| rs9536961 | G | A | G | A | 13 | -0.011759 | 0.00207 | 1.00E-200 | -0.0466 | 0.0637 | 0.4648 |
| rs982890 | C | T | C | T | 11 | 0.013959 | 0.001939 | 1.00E-200 | 0.0524 | 0.0613 | 0.3928 |
| rs9861009 | C | T | C | T | 3 | 0.012425 | 0.002185 | 1.00E-200 | 0.0725 | 0.0698 | 0.2992 |
| rs9889884 | C | T | C | T | 17 | 0.013186 | 0.002291 | 1.00E-200 | -0.1324 | 0.0799 | 0.0976697 |
| rs9926530 | G | T | G | T | 16 | 0.022162 | 0.004329 | 1.00E-200 | -0.0045 | 0.1258 | 0.9717 |
| rs9928757 | C | G | C | G | 16 | -0.013101 | 0.002423 | 1.00E-200 | 0.0723 | 0.0701 | 0.3025 |
| rs9989419 | G | A | G | A | 16 | -0.012128 | 0.001995 | 1.00E-200 | 0.0298 | 0.0628 | 0.6356 |
| rs9998874 | G | T | G | T | 4 | 0.026735 | 0.00395 | 1.00E-200 | -0.0156 | 0.139 | 0.9106 |
| rs80067728 | G | A | G | A | 4 | 0.05381 | 0.008794 | 1.00E-200 | -0.2298 | 0.2004 | 0.2515 |
| rs80204526 | A | C | A | C | 18 | -0.048218 | 0.00942 | 1.00E-200 | 0.2393 | 0.4274 | 0.5756 |
| rs897438 | G | A | G | A | 18 | -0.011957 | 0.002328 | 1.00E-200 | -0.0163 | 0.0742 | 0.8256 |
| rs942380 | G | A | G | A | 6 | 0.011434 | 0.001981 | 1.00E-200 | -0.0611 | 0.0675 | 0.3655 |
| rs951914 | C | G | C | G | 8 | -0.011397 | 0.002161 | 1.00E-200 | -0.01 | 0.0644 | 0.877 |
| rs9536961 | G | A | G | A | 13 | -0.011759 | 0.00207 | 1.00E-200 | -0.0466 | 0.0637 | 0.4648 |
| rs982890 | C | T | C | T | 11 | 0.013959 | 0.001939 | 1.00E-200 | 0.0524 | 0.0613 | 0.3928 |
| rs9861009 | C | T | C | T | 3 | 0.012425 | 0.002185 | 1.00E-200 | 0.0725 | 0.0698 | 0.2992 |
| rs9889884 | C | T | C | T | 17 | 0.013186 | 0.002291 | 1.00E-200 | -0.1324 | 0.0799 | 0.0976697 |
| rs9926530 | G | T | G | T | 16 | 0.022162 | 0.004329 | 1.00E-200 | -0.0045 | 0.1258 | 0.9717 |
| rs9928757 | C | G | C | G | 16 | -0.013101 | 0.002423 | 1.00E-200 | 0.0723 | 0.0701 | 0.3025 |
| rs9989419 | G | A | G | A | 16 | -0.012128 | 0.001995 | 1.00E-200 | 0.0298 | 0.0628 | 0.6356 |
| rs9998874 | G | T | G | T | 4 | 0.026735 | 0.00395 | 1.00E-200 | -0.0156 | 0.139 | 0.9106 |

| Supplementary table 9. MR-PRESSO estimates between vitamin D and systemic lupus erythematosus | | | | | | | |
| --- | --- | --- | --- | --- | --- | --- | --- |
| Exposure | Outcome | Model | Heterogeneity | | | Pleiotropy | |
|  |  |  | Method | Cochran’s Q | P-value | Egger-intercept(95%CI) | P-value |
| SLE | Vitamin D | Model 1 | IVW | 10.8 | 0.148 | 0.002(-0.004,0.008) | 0.396 |
| SLE | Vitamin D | Model 2 | IVW | 8.4 | 0.212 | 0.002(-0.004,0.008) | 0.523 |
| SLE | 25-hydroxyvitamin D | Model 1 | IVW | 83.5 | ＜0.001 | 0.001(-0.001,0.003) | 0.328 |
| SLE | 25-hydroxyvitamin D | Model 2 | IVW | 59.1 | 0.003 | 0.001(-0.002,0.004) | 0.707 |
| Vitamin D | SLE | Model 1 | IVW | 57.6 | 0.278 | ﹣0.004(﹣0.040,0.033) | 0.839 |
| Vitamin D | SLE | Model 2 | IVW | 44.1 | 0.51 | ﹣0.001(-0.036,0.038) | 0.952 |
| 25-hydroxyvitamin D | SLE | Model 1 | IVW | 153.6 | 0.66 | ﹣0.020(﹣0.040,0.010) | 0.06 |
| 25-hydroxyvitamin D | SLE | Model 2 | IVW | 142.4 | 0.569 | 0.018(-0.-038, 0.0016) | 0.099 |
