# Supplementary figures and images for: Is Vitamin D Deficiency the Cause or the Effect of Systemic Lupus Erythematosus: Evidence from Bidirectional Mendelian Randomization Analysis
Source: J Immunol Res. 2022 Sep 21;2022:8689777. doi: 10.1155/2022/8689777 (PMC9519279; doi:10.1155/2022/8689777)

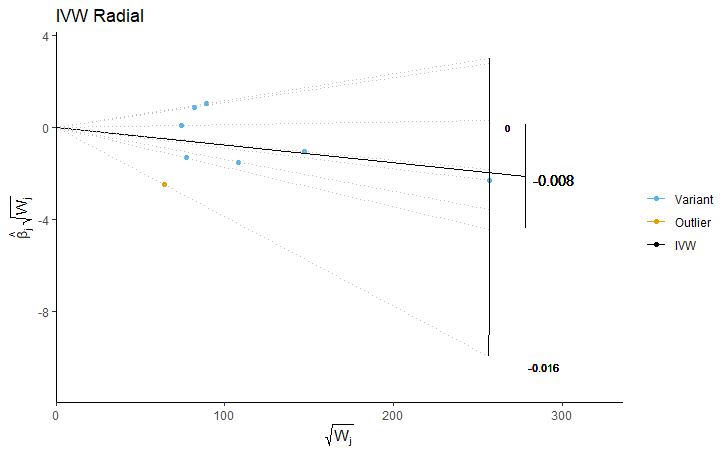

Supplement: Supplementary Materials — Supplementary table 1: harmonized dataset of univariate Mendelian randomization for the effect of SLE on vitamin D with model 1. Supplementary table 2: harmonized dataset of univariate Mendelian randomization for the effect of SLE on vitamin D with model 2. Supplementary table 3: harmonized dataset of univariate Mendelian randomization for the effect of SLE on 25-hydroxyvitamin D with model 1. Supplementary table 4: harmonized dataset of univariate Mendelian randomization for the effect of SLE on 25-hydroxyvitamin D with model 2. Supplementary table 5: harmonized dataset of univariate Mendelian randomization for the effect of vitamin D on SLE with model 1. Supplementary table 6: harmonized dataset of univariate Mendelian randomization for the effect of vitamin D on SLE with model 2. Supplementary table 7: harmonized dataset of univariate Mendelian randomization for the effect of 25-hydroxyvitamin D on SLE with model 1. Supplementary table 8: harmonized dataset of univariate Mendelian randomization for the effect of 25-hydroxyvitamin D on SLE with model 2. Supplementary table 9: MR-PRESSO estimates between vitamin D and systemic lupus erythematosus. Supplementary figure 1: IVW radial for the effect of SLE on vitamin D with model 1. Supplementary figure 2: IVW radial for the effect of SLE on vitamin D with model 2. Supplementary figure 3: diagnostic plots generated by MR-RAPS of SLE on vitamin D with model 1. Supplementary figure 4: diagnostic plots generated by MR-RAPS of SLE on vitamin D with model 2. Supplementary figure 5: IVW radial for the effect of SLE on 25-hydroxyvitamin D levels with model 1. Supplementary figure 6: IVW radial for the effect of SLE on 25-hydroxyvitamin D levels with model 2. Supplementary figure 7: IVW radial for the effect of vitamin D on SLE with model 1. Supplementary figure 8: IVW radial for the effect of vitamin D on SLE with model 2. Supplementary figure 9: diagnostic plots generated by MR-RAPS of vitamin D on SLE with model 1. Supplem [file 8689777.f1.zip › SF1 (1).png]

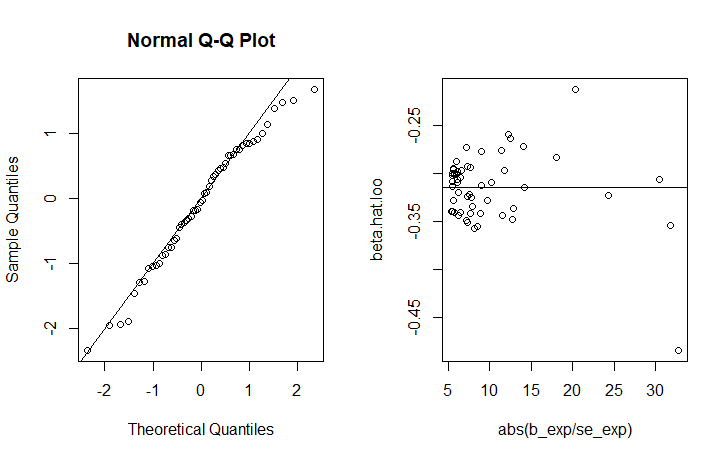

Supplement: Supplementary Materials — Supplementary table 1: harmonized dataset of univariate Mendelian randomization for the effect of SLE on vitamin D with model 1. Supplementary table 2: harmonized dataset of univariate Mendelian randomization for the effect of SLE on vitamin D with model 2. Supplementary table 3: harmonized dataset of univariate Mendelian randomization for the effect of SLE on 25-hydroxyvitamin D with model 1. Supplementary table 4: harmonized dataset of univariate Mendelian randomization for the effect of SLE on 25-hydroxyvitamin D with model 2. Supplementary table 5: harmonized dataset of univariate Mendelian randomization for the effect of vitamin D on SLE with model 1. Supplementary table 6: harmonized dataset of univariate Mendelian randomization for the effect of vitamin D on SLE with model 2. Supplementary table 7: harmonized dataset of univariate Mendelian randomization for the effect of 25-hydroxyvitamin D on SLE with model 1. Supplementary table 8: harmonized dataset of univariate Mendelian randomization for the effect of 25-hydroxyvitamin D on SLE with model 2. Supplementary table 9: MR-PRESSO estimates between vitamin D and systemic lupus erythematosus. Supplementary figure 1: IVW radial for the effect of SLE on vitamin D with model 1. Supplementary figure 2: IVW radial for the effect of SLE on vitamin D with model 2. Supplementary figure 3: diagnostic plots generated by MR-RAPS of SLE on vitamin D with model 1. Supplementary figure 4: diagnostic plots generated by MR-RAPS of SLE on vitamin D with model 2. Supplementary figure 5: IVW radial for the effect of SLE on 25-hydroxyvitamin D levels with model 1. Supplementary figure 6: IVW radial for the effect of SLE on 25-hydroxyvitamin D levels with model 2. Supplementary figure 7: IVW radial for the effect of vitamin D on SLE with model 1. Supplementary figure 8: IVW radial for the effect of vitamin D on SLE with model 2. Supplementary figure 9: diagnostic plots generated by MR-RAPS of vitamin D on SLE with model 1. Supplem [file 8689777.f1.zip › SF10.png]

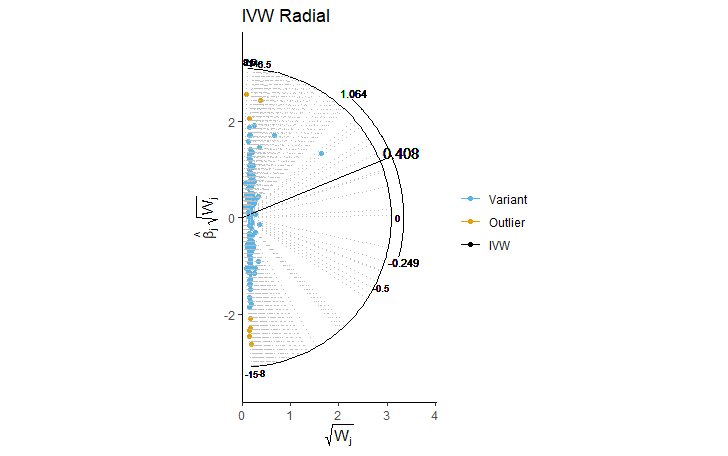

Supplement: Supplementary Materials — Supplementary table 1: harmonized dataset of univariate Mendelian randomization for the effect of SLE on vitamin D with model 1. Supplementary table 2: harmonized dataset of univariate Mendelian randomization for the effect of SLE on vitamin D with model 2. Supplementary table 3: harmonized dataset of univariate Mendelian randomization for the effect of SLE on 25-hydroxyvitamin D with model 1. Supplementary table 4: harmonized dataset of univariate Mendelian randomization for the effect of SLE on 25-hydroxyvitamin D with model 2. Supplementary table 5: harmonized dataset of univariate Mendelian randomization for the effect of vitamin D on SLE with model 1. Supplementary table 6: harmonized dataset of univariate Mendelian randomization for the effect of vitamin D on SLE with model 2. Supplementary table 7: harmonized dataset of univariate Mendelian randomization for the effect of 25-hydroxyvitamin D on SLE with model 1. Supplementary table 8: harmonized dataset of univariate Mendelian randomization for the effect of 25-hydroxyvitamin D on SLE with model 2. Supplementary table 9: MR-PRESSO estimates between vitamin D and systemic lupus erythematosus. Supplementary figure 1: IVW radial for the effect of SLE on vitamin D with model 1. Supplementary figure 2: IVW radial for the effect of SLE on vitamin D with model 2. Supplementary figure 3: diagnostic plots generated by MR-RAPS of SLE on vitamin D with model 1. Supplementary figure 4: diagnostic plots generated by MR-RAPS of SLE on vitamin D with model 2. Supplementary figure 5: IVW radial for the effect of SLE on 25-hydroxyvitamin D levels with model 1. Supplementary figure 6: IVW radial for the effect of SLE on 25-hydroxyvitamin D levels with model 2. Supplementary figure 7: IVW radial for the effect of vitamin D on SLE with model 1. Supplementary figure 8: IVW radial for the effect of vitamin D on SLE with model 2. Supplementary figure 9: diagnostic plots generated by MR-RAPS of vitamin D on SLE with model 1. Supplem [file 8689777.f1.zip › SF11.png]

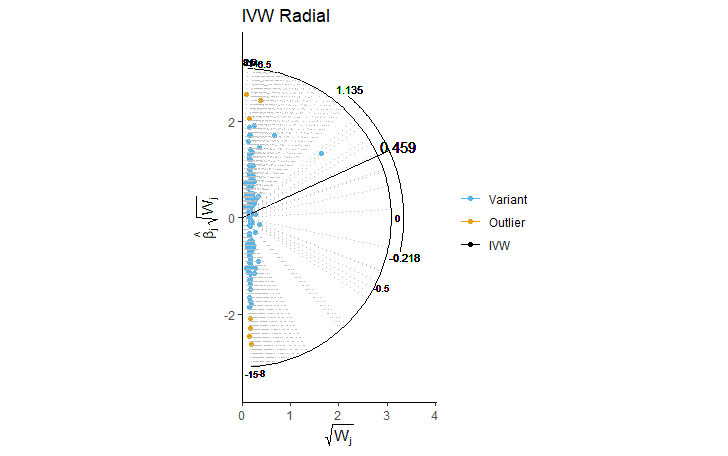

Supplement: Supplementary Materials — Supplementary table 1: harmonized dataset of univariate Mendelian randomization for the effect of SLE on vitamin D with model 1. Supplementary table 2: harmonized dataset of univariate Mendelian randomization for the effect of SLE on vitamin D with model 2. Supplementary table 3: harmonized dataset of univariate Mendelian randomization for the effect of SLE on 25-hydroxyvitamin D with model 1. Supplementary table 4: harmonized dataset of univariate Mendelian randomization for the effect of SLE on 25-hydroxyvitamin D with model 2. Supplementary table 5: harmonized dataset of univariate Mendelian randomization for the effect of vitamin D on SLE with model 1. Supplementary table 6: harmonized dataset of univariate Mendelian randomization for the effect of vitamin D on SLE with model 2. Supplementary table 7: harmonized dataset of univariate Mendelian randomization for the effect of 25-hydroxyvitamin D on SLE with model 1. Supplementary table 8: harmonized dataset of univariate Mendelian randomization for the effect of 25-hydroxyvitamin D on SLE with model 2. Supplementary table 9: MR-PRESSO estimates between vitamin D and systemic lupus erythematosus. Supplementary figure 1: IVW radial for the effect of SLE on vitamin D with model 1. Supplementary figure 2: IVW radial for the effect of SLE on vitamin D with model 2. Supplementary figure 3: diagnostic plots generated by MR-RAPS of SLE on vitamin D with model 1. Supplementary figure 4: diagnostic plots generated by MR-RAPS of SLE on vitamin D with model 2. Supplementary figure 5: IVW radial for the effect of SLE on 25-hydroxyvitamin D levels with model 1. Supplementary figure 6: IVW radial for the effect of SLE on 25-hydroxyvitamin D levels with model 2. Supplementary figure 7: IVW radial for the effect of vitamin D on SLE with model 1. Supplementary figure 8: IVW radial for the effect of vitamin D on SLE with model 2. Supplementary figure 9: diagnostic plots generated by MR-RAPS of vitamin D on SLE with model 1. Supplem [file 8689777.f1.zip › SF12.png]

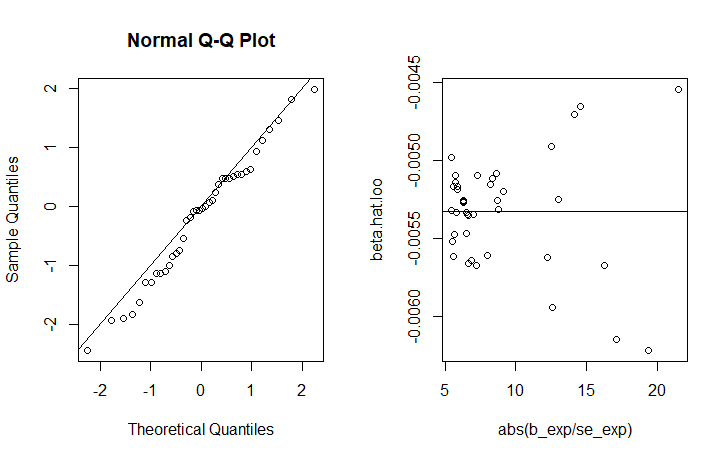

Supplement: Supplementary Materials — Supplementary table 1: harmonized dataset of univariate Mendelian randomization for the effect of SLE on vitamin D with model 1. Supplementary table 2: harmonized dataset of univariate Mendelian randomization for the effect of SLE on vitamin D with model 2. Supplementary table 3: harmonized dataset of univariate Mendelian randomization for the effect of SLE on 25-hydroxyvitamin D with model 1. Supplementary table 4: harmonized dataset of univariate Mendelian randomization for the effect of SLE on 25-hydroxyvitamin D with model 2. Supplementary table 5: harmonized dataset of univariate Mendelian randomization for the effect of vitamin D on SLE with model 1. Supplementary table 6: harmonized dataset of univariate Mendelian randomization for the effect of vitamin D on SLE with model 2. Supplementary table 7: harmonized dataset of univariate Mendelian randomization for the effect of 25-hydroxyvitamin D on SLE with model 1. Supplementary table 8: harmonized dataset of univariate Mendelian randomization for the effect of 25-hydroxyvitamin D on SLE with model 2. Supplementary table 9: MR-PRESSO estimates between vitamin D and systemic lupus erythematosus. Supplementary figure 1: IVW radial for the effect of SLE on vitamin D with model 1. Supplementary figure 2: IVW radial for the effect of SLE on vitamin D with model 2. Supplementary figure 3: diagnostic plots generated by MR-RAPS of SLE on vitamin D with model 1. Supplementary figure 4: diagnostic plots generated by MR-RAPS of SLE on vitamin D with model 2. Supplementary figure 5: IVW radial for the effect of SLE on 25-hydroxyvitamin D levels with model 1. Supplementary figure 6: IVW radial for the effect of SLE on 25-hydroxyvitamin D levels with model 2. Supplementary figure 7: IVW radial for the effect of vitamin D on SLE with model 1. Supplementary figure 8: IVW radial for the effect of vitamin D on SLE with model 2. Supplementary figure 9: diagnostic plots generated by MR-RAPS of vitamin D on SLE with model 1. Supplem [file 8689777.f1.zip › SF13.png]

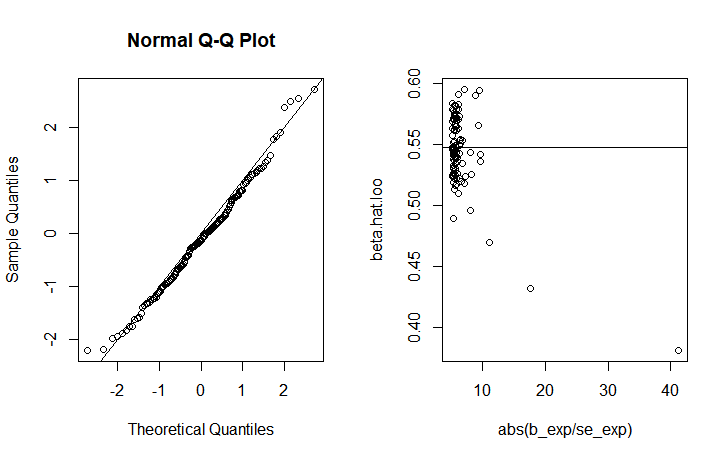

Supplement: Supplementary Materials — Supplementary table 1: harmonized dataset of univariate Mendelian randomization for the effect of SLE on vitamin D with model 1. Supplementary table 2: harmonized dataset of univariate Mendelian randomization for the effect of SLE on vitamin D with model 2. Supplementary table 3: harmonized dataset of univariate Mendelian randomization for the effect of SLE on 25-hydroxyvitamin D with model 1. Supplementary table 4: harmonized dataset of univariate Mendelian randomization for the effect of SLE on 25-hydroxyvitamin D with model 2. Supplementary table 5: harmonized dataset of univariate Mendelian randomization for the effect of vitamin D on SLE with model 1. Supplementary table 6: harmonized dataset of univariate Mendelian randomization for the effect of vitamin D on SLE with model 2. Supplementary table 7: harmonized dataset of univariate Mendelian randomization for the effect of 25-hydroxyvitamin D on SLE with model 1. Supplementary table 8: harmonized dataset of univariate Mendelian randomization for the effect of 25-hydroxyvitamin D on SLE with model 2. Supplementary table 9: MR-PRESSO estimates between vitamin D and systemic lupus erythematosus. Supplementary figure 1: IVW radial for the effect of SLE on vitamin D with model 1. Supplementary figure 2: IVW radial for the effect of SLE on vitamin D with model 2. Supplementary figure 3: diagnostic plots generated by MR-RAPS of SLE on vitamin D with model 1. Supplementary figure 4: diagnostic plots generated by MR-RAPS of SLE on vitamin D with model 2. Supplementary figure 5: IVW radial for the effect of SLE on 25-hydroxyvitamin D levels with model 1. Supplementary figure 6: IVW radial for the effect of SLE on 25-hydroxyvitamin D levels with model 2. Supplementary figure 7: IVW radial for the effect of vitamin D on SLE with model 1. Supplementary figure 8: IVW radial for the effect of vitamin D on SLE with model 2. Supplementary figure 9: diagnostic plots generated by MR-RAPS of vitamin D on SLE with model 1. Supplem [file 8689777.f1.zip › SF14.png]

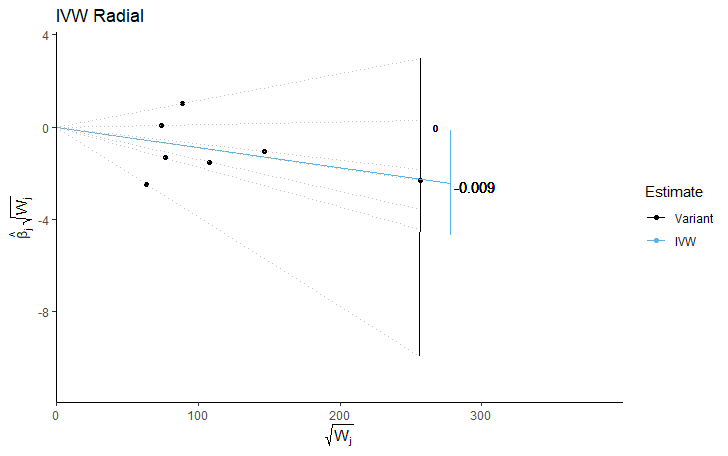

Supplement: Supplementary Materials — Supplementary table 1: harmonized dataset of univariate Mendelian randomization for the effect of SLE on vitamin D with model 1. Supplementary table 2: harmonized dataset of univariate Mendelian randomization for the effect of SLE on vitamin D with model 2. Supplementary table 3: harmonized dataset of univariate Mendelian randomization for the effect of SLE on 25-hydroxyvitamin D with model 1. Supplementary table 4: harmonized dataset of univariate Mendelian randomization for the effect of SLE on 25-hydroxyvitamin D with model 2. Supplementary table 5: harmonized dataset of univariate Mendelian randomization for the effect of vitamin D on SLE with model 1. Supplementary table 6: harmonized dataset of univariate Mendelian randomization for the effect of vitamin D on SLE with model 2. Supplementary table 7: harmonized dataset of univariate Mendelian randomization for the effect of 25-hydroxyvitamin D on SLE with model 1. Supplementary table 8: harmonized dataset of univariate Mendelian randomization for the effect of 25-hydroxyvitamin D on SLE with model 2. Supplementary table 9: MR-PRESSO estimates between vitamin D and systemic lupus erythematosus. Supplementary figure 1: IVW radial for the effect of SLE on vitamin D with model 1. Supplementary figure 2: IVW radial for the effect of SLE on vitamin D with model 2. Supplementary figure 3: diagnostic plots generated by MR-RAPS of SLE on vitamin D with model 1. Supplementary figure 4: diagnostic plots generated by MR-RAPS of SLE on vitamin D with model 2. Supplementary figure 5: IVW radial for the effect of SLE on 25-hydroxyvitamin D levels with model 1. Supplementary figure 6: IVW radial for the effect of SLE on 25-hydroxyvitamin D levels with model 2. Supplementary figure 7: IVW radial for the effect of vitamin D on SLE with model 1. Supplementary figure 8: IVW radial for the effect of vitamin D on SLE with model 2. Supplementary figure 9: diagnostic plots generated by MR-RAPS of vitamin D on SLE with model 1. Supplem [file 8689777.f1.zip › SF2.png]

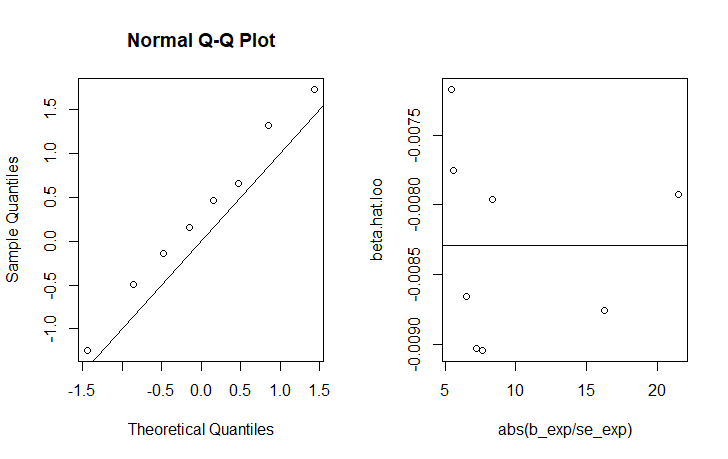

Supplement: Supplementary Materials — Supplementary table 1: harmonized dataset of univariate Mendelian randomization for the effect of SLE on vitamin D with model 1. Supplementary table 2: harmonized dataset of univariate Mendelian randomization for the effect of SLE on vitamin D with model 2. Supplementary table 3: harmonized dataset of univariate Mendelian randomization for the effect of SLE on 25-hydroxyvitamin D with model 1. Supplementary table 4: harmonized dataset of univariate Mendelian randomization for the effect of SLE on 25-hydroxyvitamin D with model 2. Supplementary table 5: harmonized dataset of univariate Mendelian randomization for the effect of vitamin D on SLE with model 1. Supplementary table 6: harmonized dataset of univariate Mendelian randomization for the effect of vitamin D on SLE with model 2. Supplementary table 7: harmonized dataset of univariate Mendelian randomization for the effect of 25-hydroxyvitamin D on SLE with model 1. Supplementary table 8: harmonized dataset of univariate Mendelian randomization for the effect of 25-hydroxyvitamin D on SLE with model 2. Supplementary table 9: MR-PRESSO estimates between vitamin D and systemic lupus erythematosus. Supplementary figure 1: IVW radial for the effect of SLE on vitamin D with model 1. Supplementary figure 2: IVW radial for the effect of SLE on vitamin D with model 2. Supplementary figure 3: diagnostic plots generated by MR-RAPS of SLE on vitamin D with model 1. Supplementary figure 4: diagnostic plots generated by MR-RAPS of SLE on vitamin D with model 2. Supplementary figure 5: IVW radial for the effect of SLE on 25-hydroxyvitamin D levels with model 1. Supplementary figure 6: IVW radial for the effect of SLE on 25-hydroxyvitamin D levels with model 2. Supplementary figure 7: IVW radial for the effect of vitamin D on SLE with model 1. Supplementary figure 8: IVW radial for the effect of vitamin D on SLE with model 2. Supplementary figure 9: diagnostic plots generated by MR-RAPS of vitamin D on SLE with model 1. Supplem [file 8689777.f1.zip › SF3.png]

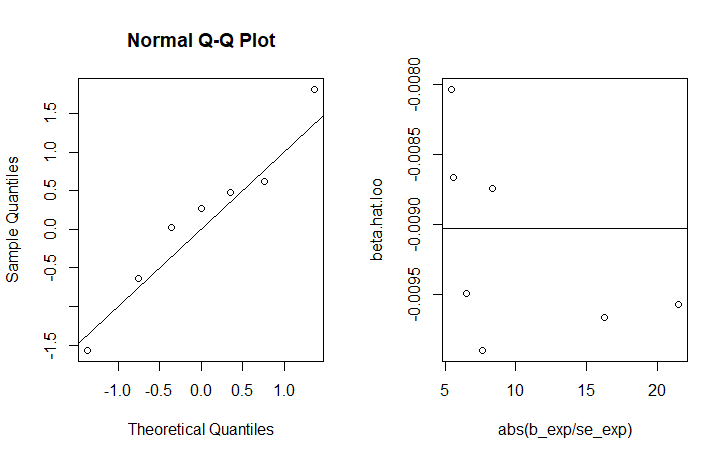

Supplement: Supplementary Materials — Supplementary table 1: harmonized dataset of univariate Mendelian randomization for the effect of SLE on vitamin D with model 1. Supplementary table 2: harmonized dataset of univariate Mendelian randomization for the effect of SLE on vitamin D with model 2. Supplementary table 3: harmonized dataset of univariate Mendelian randomization for the effect of SLE on 25-hydroxyvitamin D with model 1. Supplementary table 4: harmonized dataset of univariate Mendelian randomization for the effect of SLE on 25-hydroxyvitamin D with model 2. Supplementary table 5: harmonized dataset of univariate Mendelian randomization for the effect of vitamin D on SLE with model 1. Supplementary table 6: harmonized dataset of univariate Mendelian randomization for the effect of vitamin D on SLE with model 2. Supplementary table 7: harmonized dataset of univariate Mendelian randomization for the effect of 25-hydroxyvitamin D on SLE with model 1. Supplementary table 8: harmonized dataset of univariate Mendelian randomization for the effect of 25-hydroxyvitamin D on SLE with model 2. Supplementary table 9: MR-PRESSO estimates between vitamin D and systemic lupus erythematosus. Supplementary figure 1: IVW radial for the effect of SLE on vitamin D with model 1. Supplementary figure 2: IVW radial for the effect of SLE on vitamin D with model 2. Supplementary figure 3: diagnostic plots generated by MR-RAPS of SLE on vitamin D with model 1. Supplementary figure 4: diagnostic plots generated by MR-RAPS of SLE on vitamin D with model 2. Supplementary figure 5: IVW radial for the effect of SLE on 25-hydroxyvitamin D levels with model 1. Supplementary figure 6: IVW radial for the effect of SLE on 25-hydroxyvitamin D levels with model 2. Supplementary figure 7: IVW radial for the effect of vitamin D on SLE with model 1. Supplementary figure 8: IVW radial for the effect of vitamin D on SLE with model 2. Supplementary figure 9: diagnostic plots generated by MR-RAPS of vitamin D on SLE with model 1. Supplem [file 8689777.f1.zip › SF4.png]

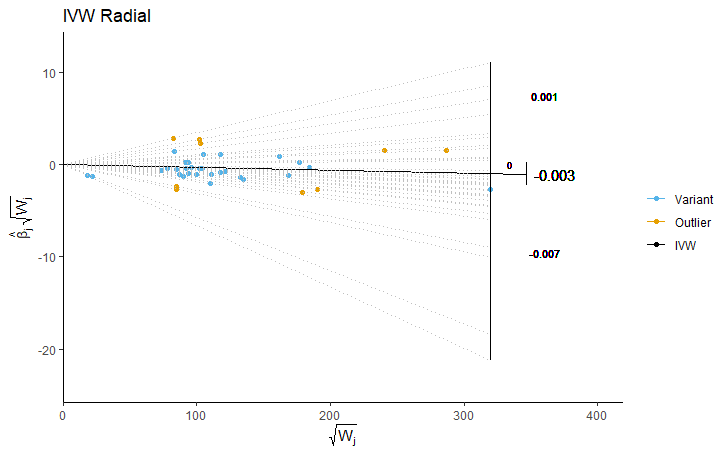

Supplement: Supplementary Materials — Supplementary table 1: harmonized dataset of univariate Mendelian randomization for the effect of SLE on vitamin D with model 1. Supplementary table 2: harmonized dataset of univariate Mendelian randomization for the effect of SLE on vitamin D with model 2. Supplementary table 3: harmonized dataset of univariate Mendelian randomization for the effect of SLE on 25-hydroxyvitamin D with model 1. Supplementary table 4: harmonized dataset of univariate Mendelian randomization for the effect of SLE on 25-hydroxyvitamin D with model 2. Supplementary table 5: harmonized dataset of univariate Mendelian randomization for the effect of vitamin D on SLE with model 1. Supplementary table 6: harmonized dataset of univariate Mendelian randomization for the effect of vitamin D on SLE with model 2. Supplementary table 7: harmonized dataset of univariate Mendelian randomization for the effect of 25-hydroxyvitamin D on SLE with model 1. Supplementary table 8: harmonized dataset of univariate Mendelian randomization for the effect of 25-hydroxyvitamin D on SLE with model 2. Supplementary table 9: MR-PRESSO estimates between vitamin D and systemic lupus erythematosus. Supplementary figure 1: IVW radial for the effect of SLE on vitamin D with model 1. Supplementary figure 2: IVW radial for the effect of SLE on vitamin D with model 2. Supplementary figure 3: diagnostic plots generated by MR-RAPS of SLE on vitamin D with model 1. Supplementary figure 4: diagnostic plots generated by MR-RAPS of SLE on vitamin D with model 2. Supplementary figure 5: IVW radial for the effect of SLE on 25-hydroxyvitamin D levels with model 1. Supplementary figure 6: IVW radial for the effect of SLE on 25-hydroxyvitamin D levels with model 2. Supplementary figure 7: IVW radial for the effect of vitamin D on SLE with model 1. Supplementary figure 8: IVW radial for the effect of vitamin D on SLE with model 2. Supplementary figure 9: diagnostic plots generated by MR-RAPS of vitamin D on SLE with model 1. Supplem [file 8689777.f1.zip › SF5.png]

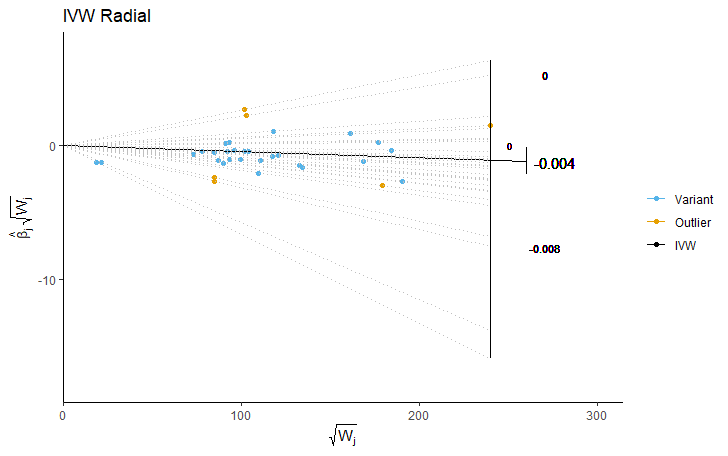

Supplement: Supplementary Materials — Supplementary table 1: harmonized dataset of univariate Mendelian randomization for the effect of SLE on vitamin D with model 1. Supplementary table 2: harmonized dataset of univariate Mendelian randomization for the effect of SLE on vitamin D with model 2. Supplementary table 3: harmonized dataset of univariate Mendelian randomization for the effect of SLE on 25-hydroxyvitamin D with model 1. Supplementary table 4: harmonized dataset of univariate Mendelian randomization for the effect of SLE on 25-hydroxyvitamin D with model 2. Supplementary table 5: harmonized dataset of univariate Mendelian randomization for the effect of vitamin D on SLE with model 1. Supplementary table 6: harmonized dataset of univariate Mendelian randomization for the effect of vitamin D on SLE with model 2. Supplementary table 7: harmonized dataset of univariate Mendelian randomization for the effect of 25-hydroxyvitamin D on SLE with model 1. Supplementary table 8: harmonized dataset of univariate Mendelian randomization for the effect of 25-hydroxyvitamin D on SLE with model 2. Supplementary table 9: MR-PRESSO estimates between vitamin D and systemic lupus erythematosus. Supplementary figure 1: IVW radial for the effect of SLE on vitamin D with model 1. Supplementary figure 2: IVW radial for the effect of SLE on vitamin D with model 2. Supplementary figure 3: diagnostic plots generated by MR-RAPS of SLE on vitamin D with model 1. Supplementary figure 4: diagnostic plots generated by MR-RAPS of SLE on vitamin D with model 2. Supplementary figure 5: IVW radial for the effect of SLE on 25-hydroxyvitamin D levels with model 1. Supplementary figure 6: IVW radial for the effect of SLE on 25-hydroxyvitamin D levels with model 2. Supplementary figure 7: IVW radial for the effect of vitamin D on SLE with model 1. Supplementary figure 8: IVW radial for the effect of vitamin D on SLE with model 2. Supplementary figure 9: diagnostic plots generated by MR-RAPS of vitamin D on SLE with model 1. Supplem [file 8689777.f1.zip › SF6.png]

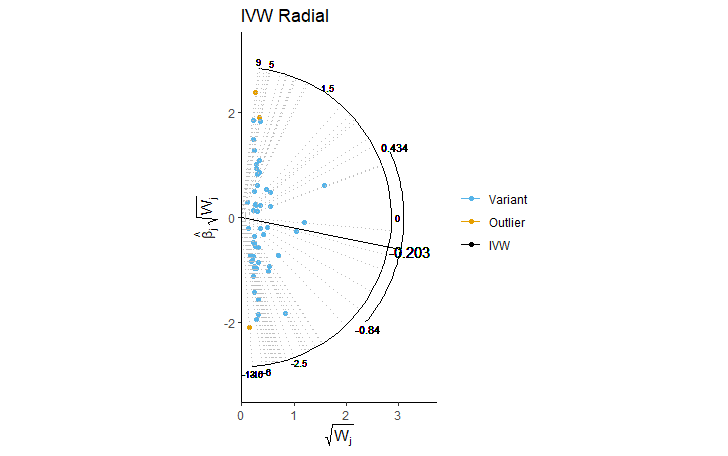

Supplement: Supplementary Materials — Supplementary table 1: harmonized dataset of univariate Mendelian randomization for the effect of SLE on vitamin D with model 1. Supplementary table 2: harmonized dataset of univariate Mendelian randomization for the effect of SLE on vitamin D with model 2. Supplementary table 3: harmonized dataset of univariate Mendelian randomization for the effect of SLE on 25-hydroxyvitamin D with model 1. Supplementary table 4: harmonized dataset of univariate Mendelian randomization for the effect of SLE on 25-hydroxyvitamin D with model 2. Supplementary table 5: harmonized dataset of univariate Mendelian randomization for the effect of vitamin D on SLE with model 1. Supplementary table 6: harmonized dataset of univariate Mendelian randomization for the effect of vitamin D on SLE with model 2. Supplementary table 7: harmonized dataset of univariate Mendelian randomization for the effect of 25-hydroxyvitamin D on SLE with model 1. Supplementary table 8: harmonized dataset of univariate Mendelian randomization for the effect of 25-hydroxyvitamin D on SLE with model 2. Supplementary table 9: MR-PRESSO estimates between vitamin D and systemic lupus erythematosus. Supplementary figure 1: IVW radial for the effect of SLE on vitamin D with model 1. Supplementary figure 2: IVW radial for the effect of SLE on vitamin D with model 2. Supplementary figure 3: diagnostic plots generated by MR-RAPS of SLE on vitamin D with model 1. Supplementary figure 4: diagnostic plots generated by MR-RAPS of SLE on vitamin D with model 2. Supplementary figure 5: IVW radial for the effect of SLE on 25-hydroxyvitamin D levels with model 1. Supplementary figure 6: IVW radial for the effect of SLE on 25-hydroxyvitamin D levels with model 2. Supplementary figure 7: IVW radial for the effect of vitamin D on SLE with model 1. Supplementary figure 8: IVW radial for the effect of vitamin D on SLE with model 2. Supplementary figure 9: diagnostic plots generated by MR-RAPS of vitamin D on SLE with model 1. Supplem [file 8689777.f1.zip › SF7.png]

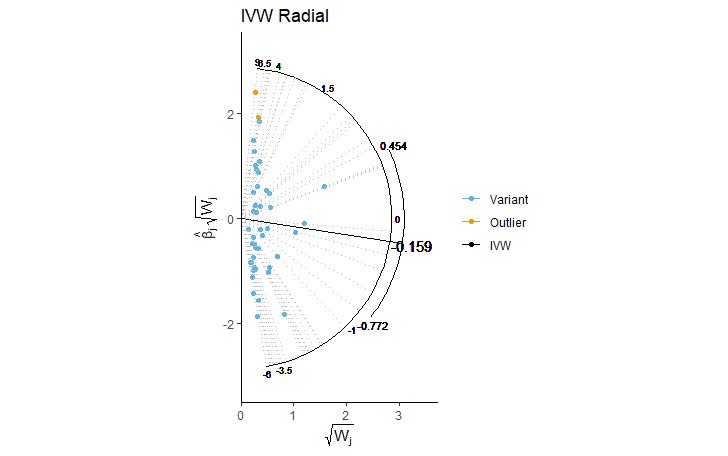

Supplement: Supplementary Materials — Supplementary table 1: harmonized dataset of univariate Mendelian randomization for the effect of SLE on vitamin D with model 1. Supplementary table 2: harmonized dataset of univariate Mendelian randomization for the effect of SLE on vitamin D with model 2. Supplementary table 3: harmonized dataset of univariate Mendelian randomization for the effect of SLE on 25-hydroxyvitamin D with model 1. Supplementary table 4: harmonized dataset of univariate Mendelian randomization for the effect of SLE on 25-hydroxyvitamin D with model 2. Supplementary table 5: harmonized dataset of univariate Mendelian randomization for the effect of vitamin D on SLE with model 1. Supplementary table 6: harmonized dataset of univariate Mendelian randomization for the effect of vitamin D on SLE with model 2. Supplementary table 7: harmonized dataset of univariate Mendelian randomization for the effect of 25-hydroxyvitamin D on SLE with model 1. Supplementary table 8: harmonized dataset of univariate Mendelian randomization for the effect of 25-hydroxyvitamin D on SLE with model 2. Supplementary table 9: MR-PRESSO estimates between vitamin D and systemic lupus erythematosus. Supplementary figure 1: IVW radial for the effect of SLE on vitamin D with model 1. Supplementary figure 2: IVW radial for the effect of SLE on vitamin D with model 2. Supplementary figure 3: diagnostic plots generated by MR-RAPS of SLE on vitamin D with model 1. Supplementary figure 4: diagnostic plots generated by MR-RAPS of SLE on vitamin D with model 2. Supplementary figure 5: IVW radial for the effect of SLE on 25-hydroxyvitamin D levels with model 1. Supplementary figure 6: IVW radial for the effect of SLE on 25-hydroxyvitamin D levels with model 2. Supplementary figure 7: IVW radial for the effect of vitamin D on SLE with model 1. Supplementary figure 8: IVW radial for the effect of vitamin D on SLE with model 2. Supplementary figure 9: diagnostic plots generated by MR-RAPS of vitamin D on SLE with model 1. Supplem [file 8689777.f1.zip › SF8.png]

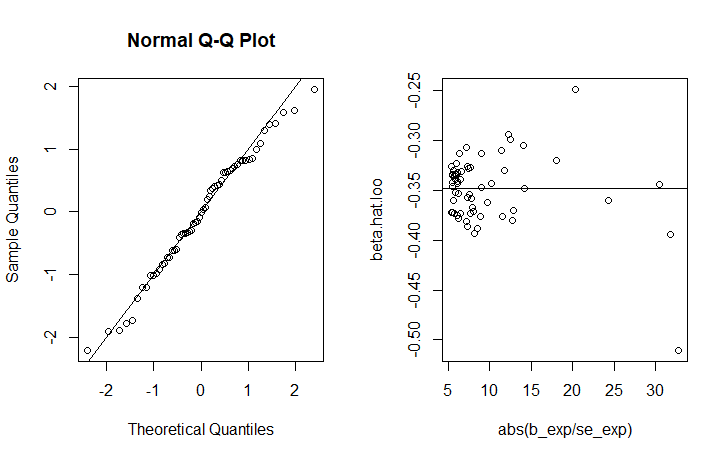

Supplement: Supplementary Materials — Supplementary table 1: harmonized dataset of univariate Mendelian randomization for the effect of SLE on vitamin D with model 1. Supplementary table 2: harmonized dataset of univariate Mendelian randomization for the effect of SLE on vitamin D with model 2. Supplementary table 3: harmonized dataset of univariate Mendelian randomization for the effect of SLE on 25-hydroxyvitamin D with model 1. Supplementary table 4: harmonized dataset of univariate Mendelian randomization for the effect of SLE on 25-hydroxyvitamin D with model 2. Supplementary table 5: harmonized dataset of univariate Mendelian randomization for the effect of vitamin D on SLE with model 1. Supplementary table 6: harmonized dataset of univariate Mendelian randomization for the effect of vitamin D on SLE with model 2. Supplementary table 7: harmonized dataset of univariate Mendelian randomization for the effect of 25-hydroxyvitamin D on SLE with model 1. Supplementary table 8: harmonized dataset of univariate Mendelian randomization for the effect of 25-hydroxyvitamin D on SLE with model 2. Supplementary table 9: MR-PRESSO estimates between vitamin D and systemic lupus erythematosus. Supplementary figure 1: IVW radial for the effect of SLE on vitamin D with model 1. Supplementary figure 2: IVW radial for the effect of SLE on vitamin D with model 2. Supplementary figure 3: diagnostic plots generated by MR-RAPS of SLE on vitamin D with model 1. Supplementary figure 4: diagnostic plots generated by MR-RAPS of SLE on vitamin D with model 2. Supplementary figure 5: IVW radial for the effect of SLE on 25-hydroxyvitamin D levels with model 1. Supplementary figure 6: IVW radial for the effect of SLE on 25-hydroxyvitamin D levels with model 2. Supplementary figure 7: IVW radial for the effect of vitamin D on SLE with model 1. Supplementary figure 8: IVW radial for the effect of vitamin D on SLE with model 2. Supplementary figure 9: diagnostic plots generated by MR-RAPS of vitamin D on SLE with model 1. Supplem [file 8689777.f1.zip › SF9.png]
